# Anti-high mobility group box-1 (HMGB1) antibody inhibits hemorrhage-induced brain injury and improved neurological deficits in rats

**DOI:** 10.1038/srep46243

**Published:** 2017-04-10

**Authors:** Dengli Wang, Keyue Liu, Hidenori Wake, Kiyoshi Teshigawara, Shuji Mori, Masahiro Nishibori

**Affiliations:** 1 Department of Pharmacology, Okayama University Graduate School of Medicine, Dentistry and Pharmaceutical Sciences, Okayama, Japan; 2 School of Pharmacy, Shujitsu University, Okayama, Japan

## Abstract

As one of the most lethal stroke subtypes, intracerebral hemorrhage (ICH) is acknowledged as a serious clinical problem lacking effective treatment. Available evidence from preclinical and clinical studies suggests that inflammatory mechanisms are involved in the progression of ICH-induced secondary brain injury. High mobility group box-1 (HMGB1) is a ubiquitous and abundant nonhistone DNA-binding protein, and is also an important proinflammatory molecule once released into the extracellular space from the nuclei. Here, we show that treatment with neutralizing anti-HMGB1 mAb (1 mg/kg, i.v. twice) remarkably ameliorated ICH-injury induced by local injection of collagenase IV in the striatum of rats. Administration of anti-HMGB1 mAb inhibited the release of HMGB1 into the extracellular space in the peri-hematomal region, reduced serum HMGB1 levels and decreased brain edema by protecting blood-brain barrier integrity, in association with decreased activated microglia and the expression of inflammation-related factors at 24 h after ICH. Consequently, anti-HMGB1 mAb reduced the oxidative stress and improved the behavioral performance of rats. These results strongly indicate that HMGB1 plays a critical role in the development of ICH-induced secondary injury through the amplification of plural inflammatory responses. Intravenous injection of neutralizing anti-HMGB1 mAb has potential as a novel therapeutic strategy for ICH.

Intracerebral hemorrhage (ICH) accounts for 10–15% of all strokes in Europe, the USA and Australia, and 20–30% of all strokes in Asia; ICH is most commonly attributed to hypertension, and is associated with extremely high rates of mortality, morbidity and disability^1^,^2^. Recently, several therapeutic targets were identified and candidate drugs were evaluated in clinical trials^3^,^4^. Unfortunately, however, there is still no effective treatment which increases survival or improves the quality of life after ICH^5^. Early surgery may limit the toxic effects of blood clot, but many clinical trials of clot evacuation in ICH have not shown a definitive benefit for surgical removal, which might reflect some of the adverse side effects of surgery^2^,^6^,^7^. ICH not only causes primary brain injury via its biochemical and mechanical effects, but also induces secondary brain injury, including local inflammatory responses to ICH and the toxic effects of blood breakdown products including hemoglobin, iron, and thrombin^1^,^4^,^8^. Secondary brain injury proceeds over hours to days, and thus it might be possible to intervene therapeutically against it^1^,^4^,^9^. However, there is also emerging evidence suggesting that inflammation contributes to brain injury during the acute phase of ICH, including breakdown of the blood–brain barrier (BBB) and activation of microglia^1^,^2^,^4^. Therefore, the suppression of inflammatory responses after ICH might be a novel strategy for reducing the secondary brain injury^2^.

High mobility group box-1 (HMGB1) is a ubiquitous and abundant nonhistone DNA-binding protein. HMGB1 is a representative of the damage-associated molecular patterns (DAMPs) family^10^, and exerts an important proinflammatory cytokine-like activity once released into the extracellular space from cellular nuclei. HMGB1 is involved in a diverse range of CNS diseases, including ischemic brain infarction, traumatic brain injury, Parkinson’s disease and neuropathic pain^11^,^12^,^13^,^14^,^15^. To trigger the inflammation, the secreted HMGB1 stimulates plural receptors i.e., the receptor for advanced glycation end products (RAGE) and toll-like receptor-2 (TLR-2) and TLR-4, which are expressed in peripheral macrophages and vascular endothelial cells as well as microglia and neurons in the central nervous system^16^,^17^. Interestingly, the administration of anti-HMGB1 neutralizing mAb has been shown to protect the BBB and to inhibit the inflammation cascade in rat models of middle cerebral artery occlusion/reperfusion-induced infarction and fluid percussion-induced traumatic brain injury^11^,^12^,^13^. The recent studies also reported the increase in HMGB1 levels in peri-hematomal regions in subacute phase after ICH in rats^18^,^19^,^20^, however, there was little information about the acute dynamics of HMGB1 in the core area after ICH. Moreover, whether anti-HMGB1 mAb can also provide neuroprotective effects in a rat model of ICH remains to be seen.

In the present study, we demonstrated that anti-HMGB1 mAb remarkably ameliorated ICH injury induced by local injection of collagenase IV in the striatum of rats, and this effect was associated with a decrease in activated microglia and astrocytes and suppression of the expression of inflammation-related factors. In addition, the treatment with anti-HMGB1 improved neurological function, which may provide a new approach to potentially reduce ongoing edema and improve the neurological outcome after ICH.

## Results

### Effects of anti-HMGB1 mAb on HMGB1 levels in the injured brain after ICH

**Figure 1:**
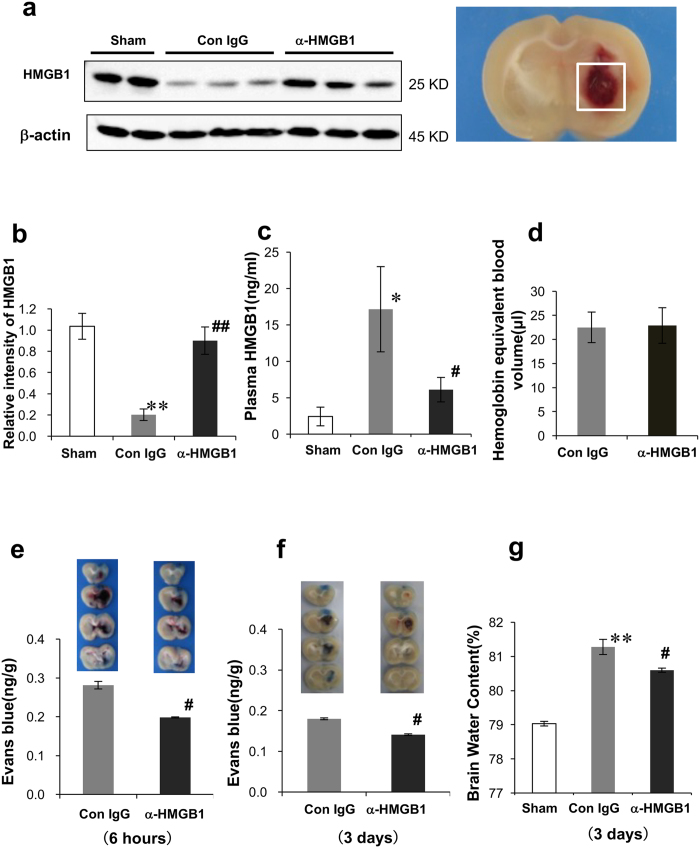
HMGB1 mobilization under ICH and effect of anti-HMGB1 mAb on HMGB1 dynamics and BBB permeability after ICH. **(a)** Cerebral bleeding was induced by injection of 0.03 U bacterial type IV collagenase into the striatum, and the resultant bleeding areas with a volume of 3 × 3 × 3 mm^3^ (as indicated by the white square in the picture) were sampled at 24 h after ICH for western blotting to determine brain HMGB1 levels. The representative results of western blotting are shown. **(b)** Quantitative analyses of the western blotting results were performed using NIH Image J software. F_(2,7)_ = 23.419, p < 0.001. **(c)** Determination of plasma levels of HMGB1 by ELISA in rats with ICH. Blood samples were collected 24 h after the induction of bleeding. F_(2,20)_ = 4.576, p = 0.023. **(d)** Evaluation of the hemorrhagic volume of rats subjected to the striatal ICH. **(e,f)** The permeability of brain capillary vessels was determined by Evans blue leakage at 6 h **(e)** and 3 days **(f)** after ICH. Representative images of Evans blue leakage at 3 h after dye injection are shown for each group. Bar graphs (lower panels) represent the results of quantification of Evans blue dye in the hemorrhagic hemisphere of the anti-HMGB1 mAb- or control IgG-treatment group. **(g)** Assessment of brain water content induced by ICH. At 3 days after ICH onset, brain water content was determined in the ipsilateral hemisphere. F_(2,9)_ = 216.059, p < 0.001. Results are shown for the sham group (Sham, n = 3, 7, 3 in (**b,c,g**), respectively), the control IgG-treated group (Con IgG, n = 3, 8, 5, 5, 5, 4 in (**b–g**), respectively), and anti-HMGB1 mAb-treated group (α-HMGB1, n = 4, 8, 5, 5, 5, 5 to (**b–g**), respectively). Values represent the means ± SEM. *p < 0.05, **p < 0.01 compared with the sham group. ^#^p < 0.05, ^##^p < 0.01 compared with the control IgG-treated group.

We confirmed that the size of the hematoma in the control and anti-HMGB1-treated rats was the same based on the measurement of hemoglobin content in each group at 24 h after ICH (Fig. 1d).

Brain striatum samples of 3 × 3 × 3 mm^3^ were excised, and HMGB1 levels were analyzed by Western blot (Fig. 1a). HMGB1 levels were decreased significantly 24 h after ICH in the control IgG-group compared with the sham group (Fig. 1b). However, anti-HMGB1 mAb administration markedly inhibited the ICH-induced decrease in HMGB1 levels compared with that in the control IgG group (Fig. 1b).

### Effects of anti-HMGB1 mAb on HMGB1 levels in the plasma after ICH

The determination of plasma levels of HMGB1 in rats treated with control IgG revealed a clear increase in HMGB1 levels at 24 h after ICH (Fig. 1c). This increase was strongly inhibited by intravenous administration of anti-HMGB1 mAb.

### Anti-HMGB1 mAb attenuates BBB disruption

We chose two time points, 6 h and 3 days after ICH, to evaluate the permeability of BBB, since it has been reported that brain edema persists for at least 3 days post-ICH^21^. Since BBB disruption very likely contributes to brain edema, changes in BBB integrity were determined by an Evans blue dye leakage assay after 6 h and 3 days, respectively. The concentration of Evans blue dye in the ipsilateral cerebrum of the control IgG group was significantly greater than that of the sham group after 6 h, indicating that ICH causes BBB disruption of the ipsilateral cerebrum (data not shown). However, the anti-HMGB1mAb group exhibited a significantly lower dye concentration than the control IgG group in the ipsilateral cerebrum at 6 h and even on day 3 post-ICH, indicating that anti-HMGB1 mAb significantly reduces the ICH-induced BBB disruption (Fig. 1e and f).

### Anti-HMGB1 mAb reduces brain edema

On day 3 post-ICH, the ipsilateral brain water content in the control mAb-treated group was 81.28 ± 0.22%, significantly larger than that in the sham group (79.03 ± 0.08%) (Fig. 1g). Anti-HMGB1 mAb significantly reduced the water content of the ipsilateral hemisphere. These findings indicate that anti-HMGB1 mAb treatment significantly reduced the cerebral edema on day 3.

### Histological studies on the effects of anti-HMGB1 mAb

**Figure 2:**
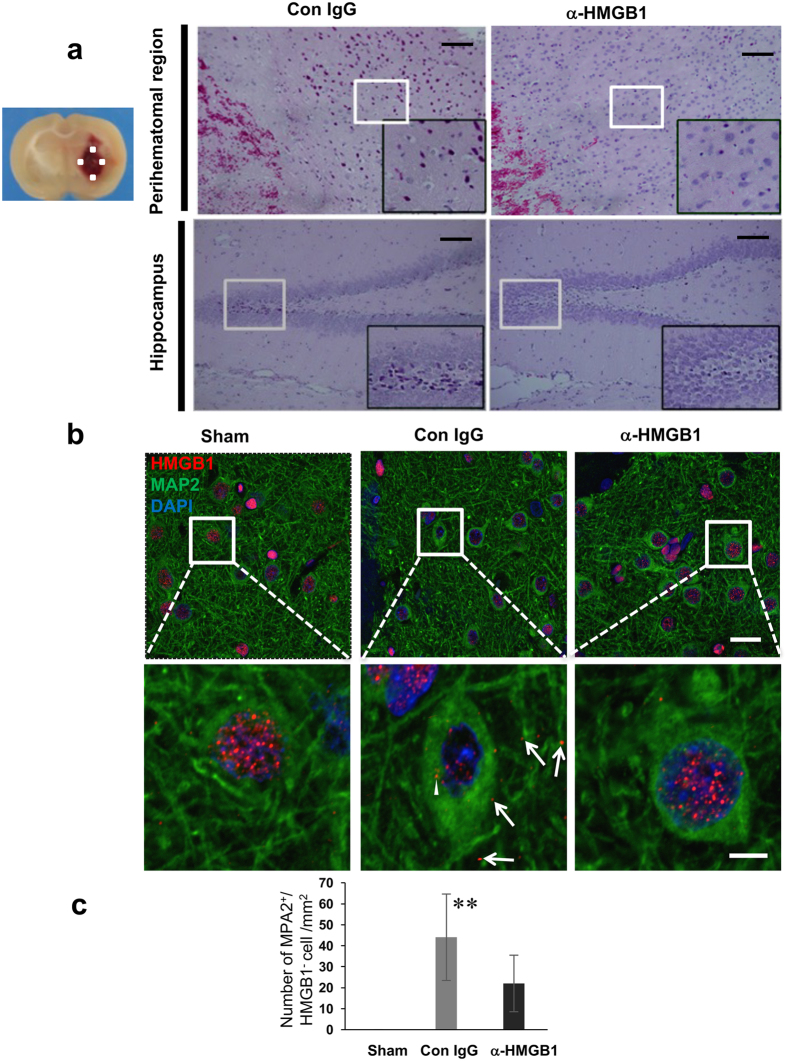
Histological evaluation of brain injury and translocation of HMGB1 in neurons 24 h after ICH. **(a)** Hematoxylin-eosin staining was performed on brain sections of rats treated with control IgG or anti-HMGB1 mAb 24 h after ICH. The fields of the ipsilateral side of the striatum (small white square) and hippocampus are shown. The left-most side of the fields from the peri-hematomal region shows the bleeding area. In the lower panels, the dentate gyrus is shown. The insets (black boxes) show magnifications of the areas of the white boxes containing many hyperchromatic cells in the control IgG-treated group. There were few such hyperchromatic cells in the anti-HMGB1- treated group. The scale bar represents 100 μm. **(b)** The rat brains were fixed at 24 h after ICH. Typical neurons in the peri-hematomal regions of the striatum as indicated in Fig. 2a are shown after the double immunohistochemical staining with anti-HMGB1 (red) and anti-MAP2 (green). Arrowheads indicate the cytosolic HMGB1. Arrows indicate the extracellular HMGB1. The lower panel shows higher magnification images of the white boxes inset in the upper-panel images. The scale bar in the lower magnification image represents 20 μm. The scale bar in the higher magnification image represents 5 μm. **(c)** The number of HMGB1 negative neurons was analyzed. The cells in which HMGB1 completely disappeared from nuclei were recognized as HMGB1-negative cells. F_(2,10)_ = 1.539, p = 0.262. Results are shown for the sham group (Sham, n = 3), the control IgG-treated group (Con IgG, n = 5), and the anti-HMGB1 mAb-treated group (α-HMGB1, n = 5). Values represent the means ± SE. **p < 0.01 compared with the sham groups.

Hematoxylin-eosin staining of brain sections from the control mAb-treated rats revealed diffuse hyperchromatic cells in the perihematoma (about 200 μm from the edge of the hematoma) and dentate gyrus in the hippocampus regions 24 h after ICH (Fig. 2a). In contrast, similar cells were rarely observed in the same areas in the rats treated with anti-HMGB1 mAb.

### Release of HMGB1 from neurons, astrocytes, and microglia

The localization of HMGB1 in the rat brain after collagenase-induced ICH was observed by immunofluorescence. As shown in previous studies^12^,^13^, in the sham group, HMGB1 was localized in the nuclear compartment of cells including neurons (Fig. 2b). In the peri-hematomal regions, the nuclear immunoreactivities of HMGB1 were decreased significantly in most of the MAP-2-positive neurons 24 h after ICH induction (Fig. 2b). HMGB1 immunoreactivities completely disappeared in a small population of neurons (Fig. 2b), while most of the neurons exhibited lower levels of HMGB1 in the nuclear compartment along with dot–like immunoreactivity in the cytosolic and extracellular compartment (Fig. 2b). The intravenous administration of anti-HMGB1 mAb significantly inhibited the above-described translocation of HMGB1 (Fig. 2b).

**Figure 3:**
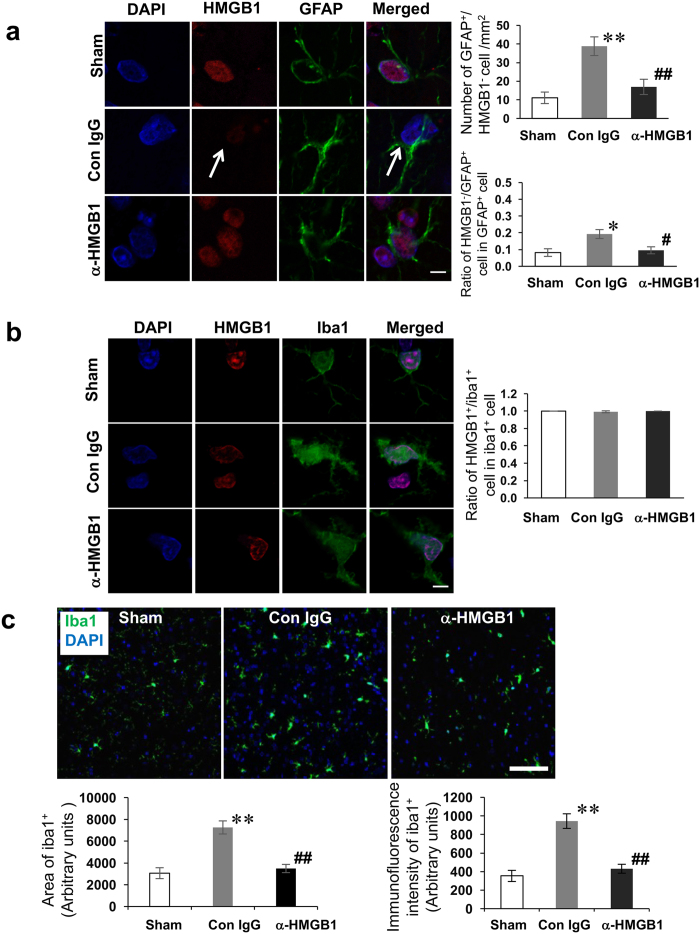
Release of HMGB1 in astrocytes and microglia and the activation of microglia 24 h after ICH. **(a)** The astrocytes in the dentate gyrus were double-immunostained with anti-HMGB1 (red) and anti-GFAP (green). Arrow indicates the HMGB1-negative astrocytes in control IgG-treated rat. The scale bar represents 5 μm. The cells in which HMGB1 completely disappeared from nuclei were recognized as HMGB1-negative cells. The number of HMGB1-negative astrocytes (F_(2,10)_ = 8.762, p = 0.006) and the ratio of these cells in total astrocytes (F_(2,10)_ = 6.944, p = 0.012) are expressed as the means ± SEM. **(b)** Typical microglia in the peri-hematomal region are shown after the double immunohistochemical staining with anti-HMGB1 and anti-Iba1. The scale bar represents 5 μm. The ratio of HMGB1-positive microglia to total microglia (F_(2,12)_ = 2.298, p = 0.143) are expressed as the means ± SEM. **(c)** The Iba1-positive cell numbers were counted at 24 h post-ICH in the peri-hematomal region of the sham, control IgG- and anti-HMGB1-treated groups. Iba1-positive cells in the control IgG-treated group had short processes and relatively larger cell bodies. The scale bar represents 100 μm. F_(2,15)_ = 20.537, p < 0.001 for area of iba1^+^ cell. F_(2,15)_ = 14.090, p < 0.001 for immunofluorescence intensity of iba1^+^ cell. Results are shown for the sham group (Sham, n = 3, 3, 5, 6, 6 in (a-c), respectively), the control IgG-treated group (Con IgG, n = 5, 5, 5, 6, 6 in (a-c), respectively), and anti-HMGB1 mAb-treated group (α-HMGB1, n = 5, 5, 5, 6, 6 to (a-c), respectively). *p < 0.01, **p < 0.01 compared with the sham group. ^#^p < 0.05, ^##^p < 0.01 compared with the control IgG-treated group.

There were two types of GFAP-positive astrocytes in peri-hematomal regions, HMGB1-negative (Fig. 3a) and HMGB1-positive cells (data not shown), suggesting that HMGB1 was released from both astrocytes and neurons. The number of HMGB1-negative astrocytes was reduced by the treatment with anti-HMGB1 (Fig. 3a). Thus, anti-HMGB1 strongly inhibited the translocation of HMGB1 in astrocytes.

In contrast to neurons and astrocytes, the immunoreactivities of HMGB1 in the nuclei of microglia in the peri-hematomal regions were retained in the control IgG-treated group, and there was no difference in the ratio of HMGB1-positive microglia against Iba1-positive cells among the sham, control IgG-treated and anti-HMGB1-treated groups (Fig. 3b). On the other hand, the number of Iba1-positive cells and the immunofluorescence intensity in a specific area were significantly increased in the control IgG-treated group compared to the sham group, suggesting that the activation of microglia largely occurred 24 h after ICH (Fig. 3c). The increase of the activation was strongly inhibited by treatment with anti-HMGB1 mAb (Fig. 3c).

### Anti-HMGB1 mAb inhibited the AQP4 levels in ICH rats

**Figure 4:**
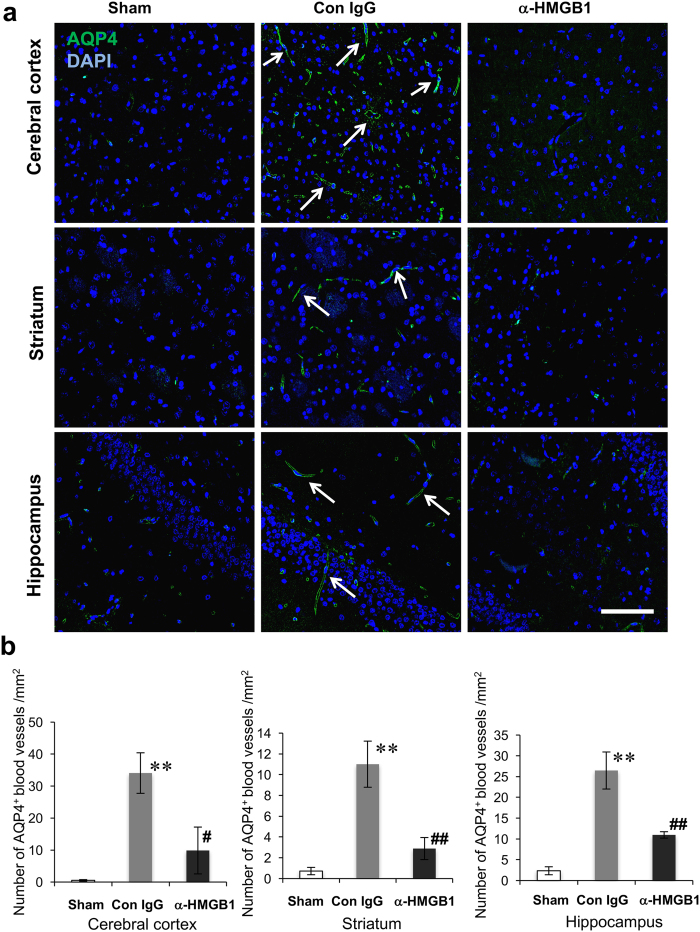
Effects of anti-HMGB1 mAb on AQP4 expression in different brain regions at 24 h after ICH. **(a)** The brains were fixed at 24 h after ICH and the brain sections were immunostained with anti-AQP4. Typical pictures of the cerebral cortex, striatum and hippocampus are shown. Arrows indicate AQP4-positive structures. The scale bar represents 100 μm. **(b)** Quantification of AQP4-immunoreactive structures was performed in the cerebral cortex (F_(2,9)_ = 15.649 p = 0.001), striatum (F_(2,9)_ = 20.575 p < 0.001) and hippocampus (F_(2,9)_ = 41.727, p < 0.001) in each group. Anti-HMGB1 mAb administration partially reversed the increase in the number of AQP4-immunoreactivities at 24 h after ICH. Results are shown for the sham group (Sham, n = 4), the control IgG-treated group (Con IgG, n = 4), and the anti-HMGB1 mAb-treated group (α-HMGB1, n = 4). Values represent the means ± SE. **p < 0.01 compared with the sham groups. ^#^P < 0.05, ^##^P < 0.01 compared with the control IgG group.

Since AQP4 expression has been suggested to be involved in increased BBB permeability, we next evaluated the effects of anti-HMGB1 mAb on AQP4 levels in ICH rats. We investigated AQP4 expression by immunohistochemistry 24 h after ICH, in the brain tissues of both control IgG- and anti-HMGB1 mAb-treated rats. As shown in Fig. 4, AQP4 immunoreactivities were observed in the cerebral cortex, striatum and hippocampus of control IgG-treated rats (white arrows in Fig. 4), whereas the immunoreactivity was much weaker in the sham group. The treatment with anti-HMGB1 led to a reduction in the immunoreactivity for AQP4 in ICH rats. We counted the number of AQP4- immunoreactive vessels (provided their lengths in the longitudinal direction were more than 10 μm) in the cerebral cortex, striatum and hippocampus. As shown in Fig. 4, the antibody-treated group showed a significantly reduced number of AQP4-positive vessels on the ipsilateral side in all these areas compared with the control IgG group.

### IL-1β induction and localization in peri-hematomal regions after ICH

**Figure 5:**
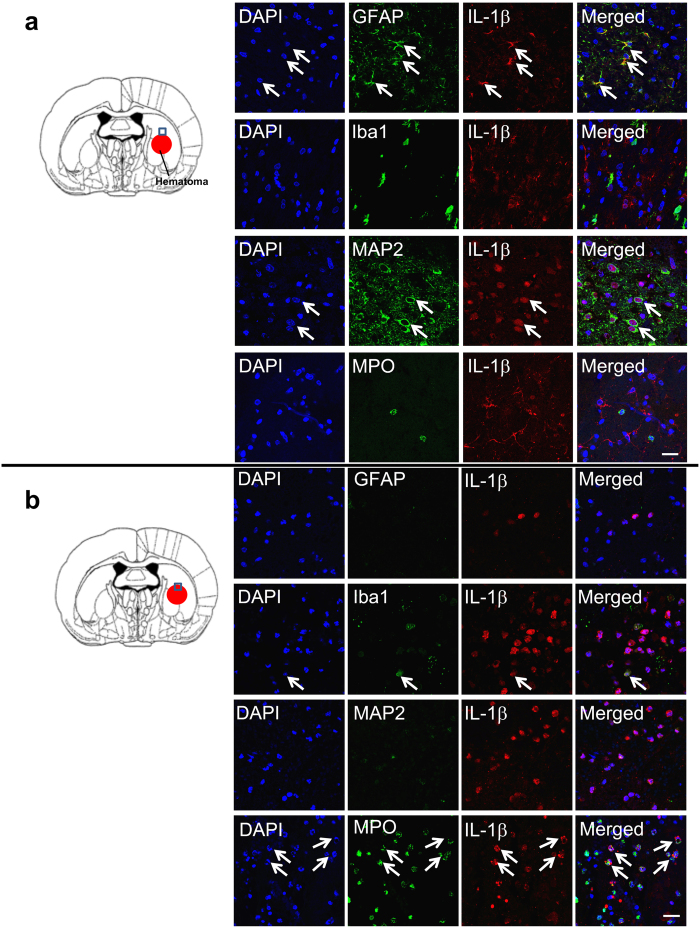
IL-1β expression and its cellular localization after ICH. **(a)** IL-1β expression in the peri-hematomal region and its cellular localization after ICH. The rat brains were fixed at 24 h after ICH and the peri-hematomal areas were double-immunostained with anti-IL-1β and anti-GFAP, anti-Iba1, anti-MAP2 or anti-MPO. IL-1β expression was co-localized with GFAP^+^ and MAP2^+^ cells, but not observed in MPO^+^ or Ibal1^+^ cells in the peri-hematomal area. White arrows show the co-localization of IL-1β with GFAP^+^ and MAP2^+^ cells, respectively. The scale bar represents 20 μm. **(b)** IL-1β expression in the central core of the bleeding area is shown. The rat brains were fixed at 24 h after ICH and the core bleeding areas were double-immunostained with anti-IL-1β and anti-GFAP, anti-Iba1, anti-MAP2 or anti-MPO. The scale bars represent 20 μm. There were almost no GFAP^+^ and MAP2^+^ cells and few iba1^+^ cells within the hematomal area. IL-1β expression was mostly co-localized with MPO^+^ cells in the peri-hematomal area. White arrows show the co-localization of IL-1β with Iba1^+^ and MPO^+^ cells, respectively.

In the present study, we observed that IL-1β immunoreactivity was almost completely restricted to reactive astrocytes and neurons in the peri-hematomal regions at 24 h after ICH (Fig. 5a). The immunoreactivity of IL-1β in astrocytes was merged with that of GFAP, whereas the immunoreactivity of IL-1β in neurons was merged with DAPI staining. These findings suggested that the IL-1β localizations in astrocytes and neurons were cytosolic and nuclear, respectively. There were no IL-1β-positive cells in the Iba1- or MPO-positive populations. We frequently observed IL-1β^+^ astrocytic processes surrounding and embracing blood vessels in the peri-hematomal region (Supplementary Fig. S5).

### IL-1β-positive cells within hematoma

Within the hematoma, the IL-1β immunoreactivity was almost exclusively limited to neutrophils based on their morphology and the MPO expression (Fig. 5b). However, only a few Iba-1^+^ microglial cells showed IL-1β expression within the hematomal region at 24 h after ICH.

### Effects of anti-HMGB1 on the expression of inflammation-related molecules and vasoconstriction-related receptors

**Figure 6:**
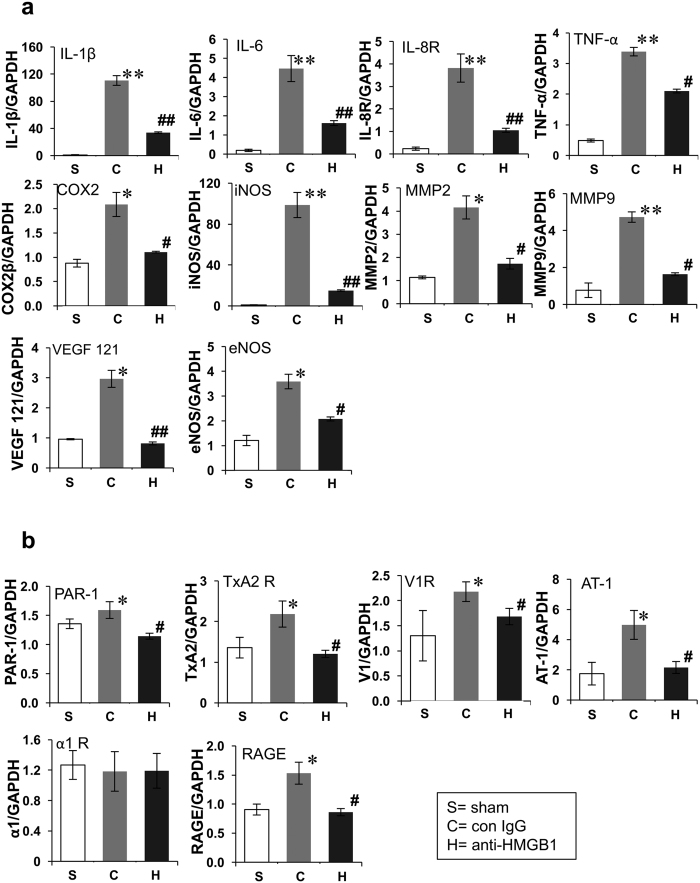
Effects of anti-HMGB1 mAb on the expression of inflammation-related and vasoconstriction-related molecules 24 h after ICH. (**a,b**) mRNA expression was measured by quantitative real-time polymerase chain reaction in the ipsilateral (injured) striatum at 24 h after ICH. The results are expressed as the means ± SEM of 4–6 rats. F value for each result was shown below as IL-6 (F_(2,13)_ = 22.775, p < 0.001), TNF-α (F_(2,13)_ = 22.460, p < 0.001), iNOS (F_(2,13)_ = 10.218, p = 0.002), IL-8R (F_(2,13)_ = 9.028, p = 0.003), IL-1β (F_(2,13)_ = 25.537, p < 0.001), COX2 (F_(2,13)_ = 5.061, p = 0.024), VEGF121 (F_(2,15)_ = 7.991, p = 0.004), MMP9 (F_(2,15)_ = 9.468, p = 0.002), MMP2 (F_(2,13)_ = 3.664, P = 0.055), α1 R (F_(2,14)_ = 0.815, p = 0.463), TxA2 (F_(2,14)_ = 7.768, P = 0.005), eNOS (F_(2,14)_ = 24.739, P < 0.001), AT-1(F_(2,13)_ = 7.901, p = 0.006), PAR1(F_(2,15)_ = 7.232, P = 0.006), V1 (F_(2,15)_ = 5.191, p = 0.019), RAGE (F_(2,15)_ = 10.928, P = 0.001). Results are shown for the sham group (Sham, n = 5–6), the control IgG-treated group (Con IgG, n = 5–6), and the anti-HMGB1 mAb-treated group (α-HMGB1, n = 6). Values represent the means ± SE. *p < 0.05, **p < 0.01 compared with the sham groups. ^#^p < 0.05, ^##^p < 0.01 compared with the control IgG-treated group.

To analyze the anti-inflammatory mechanism for the effects of anti-HMGB1 mAb, we examined the expression of inflammation- and vasoconstriction-related molecules in the peri-hematomal region of the striatum using quantitative real-time RT-PCR (Fig. 6).

The expressions of TNF-α, iNOS, IL-1β, IL-6, IL-8R, COX-2, MMP2, MMP9 and VEGF 121 were all upregulated on the ipsilateral side of the control IgG-treated rats compared with the sham group at 24 h after ICH (Fig. 6a). These upregulations were significantly suppressed by the treatment with anti-HMGB1 mAb. The expressions of the vasoconstriction-related receptors AT-1 PAR-1, V1 and TxA2 were up-regulated, and the up-regulations were suppressed by anti-HMGB1 mAb treatment (Fig. 6b). The expression of α1-adrenergic receptor was not changed after ICH (Fig. 6b). Endothelial NOS was induced in response to ICH and this induction was inhibited by anti-HMGB1 mAb (Fig. 6a).

### Inhibition of IL-1β protein expression by anti-HMGB1

**Figure 7:**
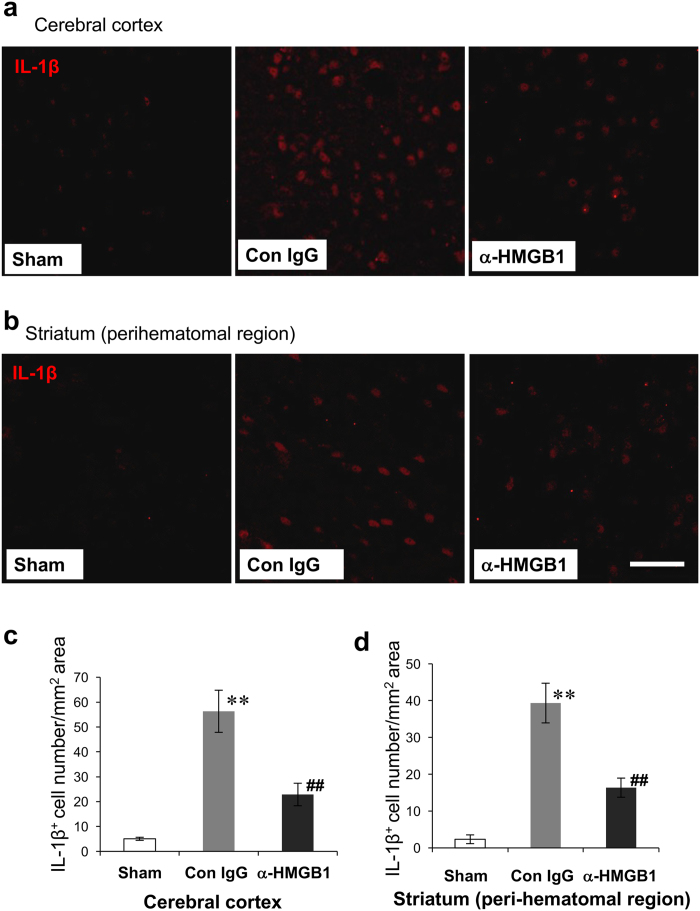
Effects of anti-HMGB1 mAb on the expression of IL-1β in the rat brain at 24 h after ICH. **(a,b)** The rat brains were fixed at 24 h after ICH. The brain sections stained with anti-IL-1β in the cerebral cortex **(a)** and peri-hematomal region in the striatum **(b)** are shown. Scale bars represent 50 μm. **(c,d)** IL-1β-positive cells were counted in the cerebral cortex (F_(2,9)_ = 26.541, p < 0.001) **(c)** and peri-hematomal striatum (F_(2,9)_ = 38.928, p < 0.001) **(d)** in each group and the results are presented as the means ± SEM. **p < 0.01 compared with the sham groups. ^##^p < 0.01 compared to the control IgG group.

The total number of IL-1β-positive cells in the ipsilateral cerebral cortex and in the peri-hematomal region was reduced significantly by the administration of anti- HMGB1 (Fig. 7). The treatment with anti-HMGB1 mAb suppressed the expression significantly. Then, we counted the IL-1β-positive cells. As shown in Fig. 7c,d, the treatment with anti-HMGB1 mAb significantly reduced the number of IL-1β-positive cells on the ipsilateral side compared with the control IgG group.

### Effect of anti-HMGB1 mAb on glial cell activation af**ter IC**H

Immunohistochemical staining of Iba1 showed microglial activation in the peri-hematomal regions after ICH. Amoeba-shaped activated microglia were found around the hematoma at 24 h after ICH (Fig. 3c). The number of Iba1-positive cells in the control mAb-treated group was significantly higher than that in the sham group. Anti-HMGB1 mAb administration significantly reduced the numbers of activated microglia compared with the control IgG group. In contrast to neurons, HMGB1 in the microglia was retained in the nuclei (Fig. 3b). The increase in the number of Iba1-positive microglia was observed not only in the peri-hematomal striatum but also the cerebral cortex, corpus callosum and hippocampus (Supplementary Fig. S1a and b).

### Anti-HMGB1 mAb decreased oxidative stress

**Figure 8:**
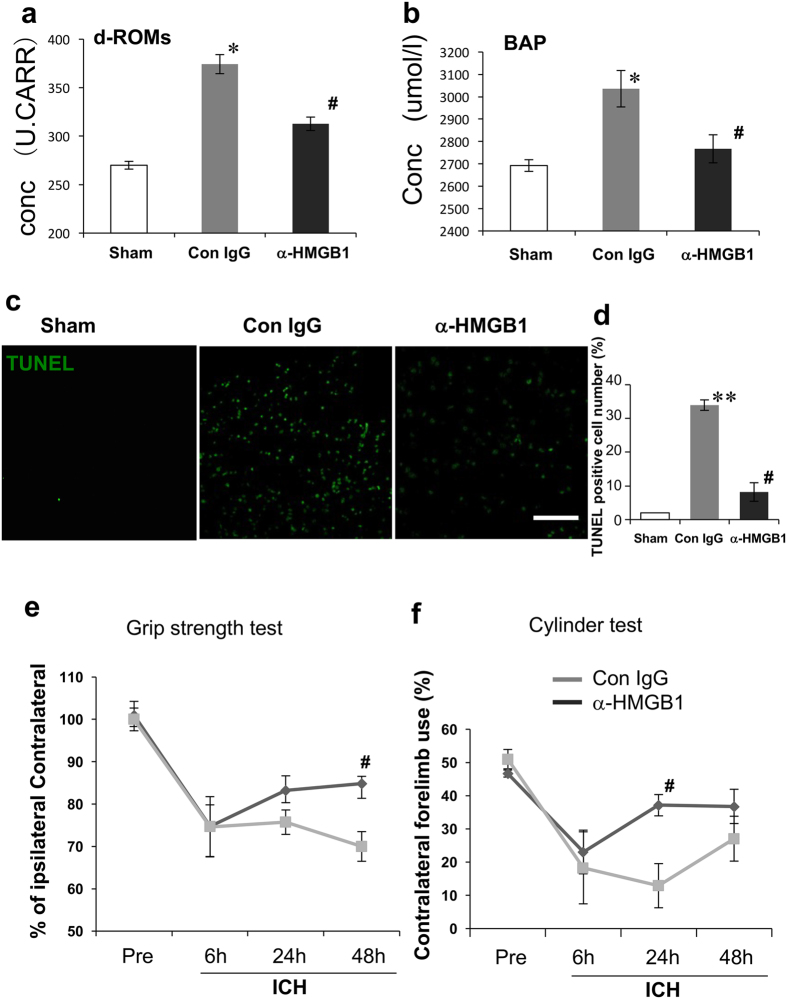
Effects of anti-HMGB1 mAb on oxidative stress, cell apoptosis and the impairment of motor functions after ICH. **(a)** The blood samples were collected from the left ventricle of the heart at 24 h after ICH. Diacron-reactive oxygen metabolites (d-ROM) were measured using serum samples. F_(2,20)_ = 10.953, p < 0.001. **(b)** Changes in the serum bioantioxidant potentials (BAP) at 24 h after ICH. BAP was measured using serum samples. F_(2,20)_ = 3.833, p = 0.039. **(c,d)** TUNEL staining to reveal apoptotic cells in the ipsilateral striatum in each group. The scale bar represents 100 μm. F_(2,9)_ = 50.182, p < 0.001. **(e)** The grip strength test of contralateral forelimb was measured at the indicated times in each rat as described in the Methods section. A hundred % represents the pre-experiment strength. F_(7,36)_ = 9.867, p < 0.001. **(f)** Contralateral forelimb use in the cylinder tested at the indicated times after ICH. F_(7,36)_ = 4.222, p = 0.002. Results are shown for the sham group (Sham, n = 7, 7, 4 in (**a,b,d**), respectively), the control IgG-treated group (Con IgG, n = 8, 8, 4, 5, 5 in (**a,b,d,e,f**), respectively), and anti-HMGB1 mAb-treated group (α-HMGB1, n = 8, 8, 4, 6, 6 to (**a,b,d,e,f**), respectively). Values represent the means ± SEM. *p < 0.05, **p < 0.01 compared with the sham group. ^#^p < 0.05 compared with the control IgG-treated group.

It is well known that ROS are produced during normal oxidative metabolism, but high ROS levels may damage neurons and cause neuronal death. The serum hydroperoxide concentration was determined and the results are shown in Fig. 8a. Twenty-four hours after ICH, the hydroperoxide concentration increased in the control IgG-treated groups. However, the levels in the anti-HMGB1 group remained significantly lower than that in the control group. To evaluate the oxidant/antioxidant balance after ICH, the serum bioantioxidant potency (BAP) was measured (Fig. 8b). The pattern of serum bioantioxidant potency was roughly parallel to that of the ROS. When comparing the two groups, the BAP in the control group was significantly higher than that in the anti-HMGB1-treated group.

### TUNEL-positive cells

We evaluated ICH-induced cell death in the peri-hematomal regions using TUNEL-staining. The TUNEL-positive apoptotic cells were observed in the ipsilateral striatum in the control IgG-treated rats (Fig. 8c). The number of TUNEL-positive cells at 24 h was much higher than that in sham rats. Few TUNEL-positive cells were observed in the contralateral brain in any of the three groups. The number of TUNEL-positive cells in the ipsilateral brain in the anti-HMGB1 group at 24 h was significantly lower than that in the ICH group, but significantly higher than that in the sham group (Fig. 8d).

### Anti-HMGB1 mAb improved neurological deficits after ICH

To examine the effects of anti-HMGB1 mAb on neurobehavioral deficits after ICH, we assessed the neurologic function at baseline and at 6, 24, and 48 h after ICH. Compared with the baseline values, neurologic deficits were apparent in all rats at 6 h after ICH. Rats treated with anti-HMGB1 mAb showed a significant improvement in their recovery from initial deficits. In the grip strength test, the neurological score was decreased 6 h after the induction of ICH and remained declined over the 48-h observation period in the control IgG-treated group. Treatment with anti-HMGB1 mAb significantly facilitated the recovery, and the grip strength at 48 h was significantly higher than that in the control IgG-treated group (Fig. 8e). Anti-HMGB1 antibody was also effective in alleviating the deficit in performance in the cylinder test at 24 and 48 h after the induction of ICH (Fig. 8f).

### The effects of anti-HMGB1 mAb treatment with regard to the therapeutic time window

To examine the therapeutic time window for the effects of anti-HMGB1 mAb on ICH-induced brain injury, we started the first injection of anti-HMGB1 3 hours after the onset of ICH and determined the changes in HMGB1 contents and mRNA expression of inflammation-related molecules. The results were shown in Supplementary Fig. S6. In fact, we observed a prevention of HMGB1 decrease and inhibition of mRNA expression of inflammation-related molecules including IL-1β, iNOS, IL-8R, IL-6, TNF-α, VEGF 121, TLR-2 and TLR-4 by anti-HMGB1 similar to those obtained by immediate treatment with anti-HMGB1 mAb, indicating a therapeutic time window of 3 hours in this ICH model at least.

## Discussion

In this study, we used an ICH model induced by local injection of collagenase IV into the striatum of rats to mimic spontaneous clinical ICH. Our results indicated that administration of anti-HMGB1 mAb inhibited (1) HMGB1 translocation and release, (2) brain edema, (3) microglia activation, (4) mRNA expression of pro-inflammatory cytokines, and (5) apoptotic cell death in the peri-hematomal areas, and improved the neurological performance of rats suffering from ICH. Anti-HMGB1 mAb also reduced the plasma levels of HMGB1. All of these results support the view that HMGB1 was released mainly from neuronal nuclei and partly from astrocytes in the peri-hematomal areas in the acute phase of ICH, yielding a diverse range of secondary inflammation responses that was inhibited by anti-HMGB1 mAb. As a result, the treatment with anti-HMGB1 mAb ameliorated the neurological symptoms. To the best of our knowledge, these findings are the first to demonstrate that intravenous treatment with a neutralizing anti-HMGB1 mAb had neuroprotective effects on this ICH model.

A recent clinical study reported that the serum levels of HMGB1 were dramatically increased in patients with acute ICH, and this increase was significantly correlated with stroke severity^22^. In related studies, ICH animal models were used to evaluate two pharmacological inhibitors of HMGB1 secretion, ethyl pyruvate and glycyrrhizin^17^,^23^. The administration of ethyl pyruvate improved the functional outcome, reduced brain edema and decreased the number of apoptotic cells in the rat intracerebral hemorrhage and rat traumatic brain injury models^23^,^24^. Similarly, glycyrrhizin suppressed brain edema and improved behavioral performance in ICH rats, probably by binding to HMGB1 and thereby inhibiting the interaction between HMGB1 and RAGE^17^,^25^. Together, these findings suggest that HMGB1 may be one of the key inflammatory factors in ICH pathology and that therapeutic targeting for HMGB1 produces beneficial effects in ICH. In the present study, anti-HMGB1 mAb was shown to bind to HMGB1 directly and suppress the secondary brain injury efficiently. Thus, administration of intravenous anti-HMGB1 mAb may be a quite effective therapy for ICH in addition to brain ischemia, brain trauma, and neuropathic pain^11^,^12^,^13^,^15^,^26^.

In the previous investigations on ischemic brain injury and traumatic brain injury, the translocation and release of HMGB1 from the nuclei to extracellular space through the cytosolic compartment occurred mainly in neurons^11^,^12^,^13^. Similarly, in a Parkinson’s disease model, HMGB1 was found to translocate from the nucleus to the cytoplasm and then to the extracellular space in neurons and astrocytes at days 1 and 7 post-lesion, respectively^14^. Consistent with previous studies, we also observed the translocation of HMGB1 into the cytoplasm not only in neurons but also in astrocytes in an ICH model at 24 h after bleeding. In addition, our western blotting and ELISA analyses showed that the level of HMGB1 was significantly decreased in the ipsilateral striatum (the core area of bleeding), and somewhat increased in the serum, respectively. This suggested that HMGB1 was released into the bloodstream from the damaged brain area. Lei *et al*. found elevated HMGB1 levels in the peri-hematomal region at 24 h and 72 h after ICH by western blotting^23^. The apparent discrepancy of HMGB1 dynamics between these previous studies and our present experiments may be ascribable to the difference in sampling areas and lesion size. In the present study, we sampled the hematoma core for the determination of HMGB1 with a relatively small amount of surrounding tissue, while Lei *et al*. collected the peri-hematomal region for analysis. Accordingly, we speculated that the most severe inflammation mediated by HMGB1 might occur in the center of hemorrhage due to the massive release and disappearance of HMGB1 occurring within the hematomal area.

Three receptors of HMGB1 (RAGE, TLR-2 and TLR-4) have been reported in earlier studies^16^,^27^,^28^. Although ICH was shown to significantly up-regulate the expression of all three receptors of HMGB1, only treatment with the RAGE antagonist (FPS-ZM1) significantly reduced ICH-induced infiltration of inflammatory cells and the expressions of IL-1β and MMP-9 in the peri-hematomal region^25^,^29^. In contrast, TLR2/4 antagonists had no influence on the ICH-induced infiltration of inflammatory cells, even though they did slightly reduce the expressions of IL-1 and MMP-9^25^. In the present experiments, the mRNA level of RAGE was also decreased by treatment with anti-HMGB1 mAb after ICH. Taken together, these results suggest that RAGE may be the predominant receptor for HMGB1 in the pathogenesis of inflammation after ICH, and anti-HMGB1 mAb might exert its effects through the HMGB1/RAGE signaling pathway.

Cytokines can increase the production of other cytokines via a positive feedback loop. For example, HMGB1 acting as an early pro-inflammatory cytokine promotes the production of many cytokines during ischemic brain injury, including IL-1β and TNF-α^30^. Conversely, increased levels of cytokines such as IL-1β and TNF-α have also been shown to stimulate HMGB1 secretion in different types of cells in *in vitro* experiments^31^. Here, we reported an up-regulated mRNA expression of proinflammatory molecules such as TNF-α, IL-1β, IL-6, IL-8R, iNOS and COX-2 in the peri-hematomal region after ICH induction, and a significant reduction in the expression of these molecules in the peri-hematomal region at 24 h after intravenous injection of anti-HMGB1 mAb. Hence, HMGB1 release may be one of the causative factors to enhance the expression of these proinflammatory molecules after ICH. Up-regulation of these inflammatory molecules probably contributes to astroglial proliferation and neutrophil recruitment in the region surrounding the hematoma, and causes BBB damage and brain edema as in cerebral ischemia, traumatic brain injury, and neurodegenerative disorders^32^,^33^,^34^. In addition, a recent study showed that IL-1β was bound to HMGB1 with high affinity and the association of HMGB1 with IL-1β further potentiated the inflammatory responses, as the cellular activation induced by the HMGB1-IL-1β complex was significantly greater than that found with equivalent amounts of IL-1β or HMGB1 alone^31^. In regard to the protein expression of IL-1β, we clearly showed that it was strongly up-regulated in control ICH rats, and that this up-regulation was considerably inhibited by anti-HMGB1 mAb therapy. It is therefore possible that anti-HMGB1 mAb, which has the ability to neutralize released HMGB1, diminished the synergistic proinflammatory effects between HMGB1 and IL-1β. Interestingly, it has been reported that IL-1β is expressed in the astrocytic processes surrounding and embracing blood vessels in the peri-hematomal region (Fig. S5). In addition, COX-2 inhibition treatment by celecoxib can reduce the infiltration of inflammatory cells and brain edema, resulting in a reduction of TUNEL-positive peri-hematomal cell death^35^. Notably, anti-HMGB1 mAb therapy may suppress plural pathways in the secondary inflammatory responses after ICH.

Blood components (e.g., thrombin, hemoglobin, iron) and the inflammatory responses they induce play a major role in ICH-induced BBB dysfunction^8^. Earlier studies showed up-regulation of MMP-2 and MMP-9, as well as acute brain injury, after ICH^36^. Although collagenase IV is itself one of the MMPs, it has no effect on the expression of other MMPs^37^. These findings suggest that the increase in MMP-2 and MMP-9 after ICH in our study probably contributed to further BBB disruption after collagenase. Indeed, the synthetic inhibitors of MMPs (BB-1101 and GM6001) have been shown to reduce BBB permeability and hemorrhage after ICH in animal models^8^. Here, we demonstrated that anti-HMGB1 mAb achieved a considerable reduction in collagenase IV-induced BBB disruption in the striatum, in association with a decrease of MMP2/9 expression. In addition, the mRNA expression of pro-inflammatory molecules (TNF-α, IL-1β, IL-6, IL-8R, iNOS and COX-2) and the protein expression of IL-1β were significantly increased after ICH induction. The increased levels of these molecules should be associated with a breakdown of BBB and recruitment of neutrophils into the CNS^38^,^39^. Since our previous investigations using an *in vitro* BBB system clearly demonstrated that recombinant human HMGB1 increased the vascular permeability of BBB in association with morphological changes in endothelial cells and pericytes^12^, in the present study, HMGB1 probably facilitated BBB disruption both directly and indirectly through induction of the expression of cytokines, MMPs and chemokines.

In conclusion, our findings lend support to the idea that HMGB1 contributes to ICH-induced secondary inflammatory responses, which in turn cause the microglial activation, cytokine expression and BBB disruption. Our results also suggest that anti-HMGB1 mAb could be a valuable neuroprotective agent for the treatment of ICH, even if the treatment is initiated at 3 h after the onset of hemorrhage. We propose that anti-HMGB1 mAb treatment could be a novel therapeutic strategy applicable for three types of stroke: ICH, brain infarction^11^,^12^ and subarachnoid hemorrhage^40^.

## Materials and Methods

### Animals and treatment groups

All procedures for these experiments was approved by the Committee on Animal Studies of Okayama University and carried out following the guidelines of Okayama University for animal studies. Male Wistar rats (250–300 g, n = 150) were randomly divided into 3 groups: (1) an anti-HMGB1mAb group, in which ICH injury was induced by local injection of collagenase IV in the striatum and then the rats were treated with i.v. injection of anti-HMGB1mAb; (2) a class-matched control mAb group, in which ICH injury was induced and then the rats were treated with class-matched control mAb; and (3) a sham group, in which saline was injected into the striatum instead of collagenase IV and the rats were treated with saline.

### Surgical induction of ICH and mAb administration

Rats were placed in a stereotaxic frame after anesthesia with isoflurane. Intracerebral hemorrhage was induced by the local injection of collagenase IV in the striatum of the rats, as described previously^41^. Briefly, a 30-gauge needle was inserted into the right striatum, 0.2 mm anterior to the coronal suture, 3 mm lateral to the bregma and 6 mm below the skull. Bacterial type IV collagenase (0.03 U) in 2 μl saline was delivered at a constant rate of 0.5 μl/min. The needle was left in place for an additional 5 min to prevent backflow. In the sham group, we performed the same procedures, except that we used saline injection instead of collagenase IV. Reproducible lesions and hemorrhage were produced in the striatum by this procedure (Fig. 1). After injection, we removed the needle, filled the burr hole with bone wax and sutured the wound. None of the rats died during the experiments.

The rats were administered anti-HMGB1 mAb (#10–22, IgG2a subclass, 1 mg/kg) or anti-K*eyhole Limpet hemocyanin* (class-matched control mAb, 1 mg/kg) through the tail vein immediately and 6 h after ICH induction.

### Spectrophotometric assay for hemoglobin

Brain hemorrhage was detected by quantifying the hemoglobin content with Drabkin’s reagent (Sigma, St. Louis, MO) as described previously^42^,^43^. Briefly, after removing the blood through transcardial perfusion, the ipsilateral brain tissue containing the hemorrhage region was obtained at 24 h after ICH, and PBS (3 ml) was added to each hemisphere, followed by homogenization for 1 min, sonication on ice with an ultrasonicator for 30 seconds, and centrifugation at 15,000 g for 30 min. The supernatant (40 μl) was reacted with 160 μl of Darbkin’s solution for 15 min. Fifteen minutes later, the optical density (OD) was measured at 540-nm wavelength. To derive the standard curve, aliquots of 0 μl, 10 μl, 20 μl, 30 μl, 40 μl, and 50 μl blood were added into the normal brain hemisphere, and the OD values were measured as a reflection of the amount of hemoglobin present. The OD values of the ICH-induced brains were then compared with this standard curve to obtain the data on hemorrhage volume.

### Western blot analysis

Brain samples were collected from the striatum hematoma core (3 × 3 × 3 mm^3^) and homogenized for 5 min in RIPA lysis buffer that contained a cocktail of protease inhibitors (Sigma, St. Louis, MO). The protein samples were homogenized and separated by 12% SDS–polyacrylamide gel electrophoresis, then transferred onto nitrocellulose membranes. After blocking with 10% skim milk, the membranes were incubated at 4 °C overnight with polyclonal rabbit anti-HMGB1 antibody labeled with horseradish peroxidase (made by our laboratory). β-actin, as a reference protein, was probed with a mouse anti-β-actin mAb (Santa Cruz Biotechnology, Santa Cruz, CA) followed by goat anti-mouse Ab. Finally, the bands were visualized using an ECL system (Thermo Fisher Scientific Inc., Bend, OR), and analyzed by Image J software (NIH).

### Enzyme-linked immunosorbent assay

To determine HMGB1 levels in plasma, blood samples (1 ml) were collected through the rat heart under deep anesthesia, then centrifuged for 15 min at 800 g. HMGB1 was detected by using an ELISA kit (Shino-Test Co, Sagamihara, Japan), according to the manufacturer’s instructions.

### Evaluation of BBB integrity

To evaluate BBB permeability, Evans blue extravasation was measured as described previously^44^. Briefly, 2% Evans blue dye (2 ml/kg) was injected intravenously at 6 h or 3 days after ICH. Three hours later the rats were perfused with 100 ml of saline via the left ventricle of the heart under deep anesthesia with pentobarbital sodium. The brains were immediately removed and the ipsilateral hemispheres were homogenized in 1 M potassium hydroxide. Then the homogenized mixture was placed in 50% trichloroacetic acid and centrifuged at 800 g for 30 min. The absorbance of the supernatant solution was measured at 620 nm. The levels of Evans blue (ng/g) were calculated by means of a standard curve.

### Brain water content

Brain edema of the ipsilateral brains was determined by the wet–dry weight ratio method at 72 h after ICH, as described previously^12^. Brain water content was calculated as [(wet weight − dry weight)/wet weight] × 100%.

### Immunohistochemical studies

For immunofluorescence staining, paraffin-embedded brain sections were prepared as described in our previous studies^11^. The primary antibodies used in the experiments included anti-HMGB1 Ab (R&D Systems Inc., Minneapolis, MN), anti-AQP4 Ab (Abcam Plc, Cambridge, UK), anti-IL-1β Ab (R&D Systems Inc.), anti-microtubule-associated protein 2 (MAP2) Ab (Abcam Plc), anti-glial fibrillary acid protein (GFAP) Ab (Abcam Plc), anti-ionized calcium-binding adaptor molecule 1 (Iba1) Ab (Wako, Osaka, Japan) and anti-myeloperoxidase (MPO) Ab (Abcam Plc). In order to investigate the cellular source as well as the localization of IL-1β, double immunohistochemical staining was carried out with cell marker antibodies including MAP2, GFAP, iba1 or MPO antibodies and an antibody against IL-1β. The sections were then incubated with secondary Abs conjugated with Alexa-488, Alexa-555 or Alex594, which were purchased from Invitrogen (Tokyo, Japan). Finally, the sections were mounted using VECTASHIELD Hard Set Mounting Medium with DAPI (Vector Laboratories Inc., Burlingame, CA) and observed under an LSM 780 confocal microscopic system (Carl Zeiss Inc., Jena, Germany). The counting was performed in a blinded manner.

### Quantitative real-time PCR

Real-time polymerase chain reaction (PCR) was performed as described previously^13^. The brain samples for RT-PCR were obtained from peri-hematomal regions (each sample weight about 25 mg). The primers used for the analysis of mRNA expression are summarized in Supplementary Table S1. The expressions of the following molecules were determined: interleukin-1β (IL-1β), IL-8R, tumor necrosis factor-α (TNF-α), inducible nitric oxide synthase (iNOS), matrix metalloproteinase (MMP)-2, MMP-9, vascular endothelial growth factor (VEGF)-A189, VEGF-A121, cyclooxygenase (COX)-2, angiotensin type 1 receptor (AT-1R), protease-activated receptor-1 (PAR-1), α1 adrenoceptor (α-1R), vasopressin receptor 1 (V1R), endothelial nitric oxide synthase (eNOS), thromboxane A_2_ receptor (TxA2), receptor for advanced glycation end products (RAGE) and glyceraldehyde-3-phosphate dehydrogenase (GAPDH). GAPDH expression was used as an internal control to normalize cDNA levels. Fold changes in expression levels were calculated by the comparative cycle threshold method (2^−ΔΔCT^).

### Assay of reactive oxygen metabolites and antioxidant potency (biological antioxidant potency)

Derivatives of reactive oxygen metabolites (d-ROM) and biological antioxidant potency (BAP) were measured in plasma at 24 h after ICH using a free radical electron evaluator (FREE; Health & Diagnostic Limited Co., Naples, Italy) according to the manufacturer’s instructions. In brief, the plasma was added to an acetate buffer with FeCl_2_ at 37 °C, followed by the addition of a chromogenic mixture including aromatic alkyl-amine. After incubation at 37 °C for 5 min, the colored radical derivative was measured at 505 nm. The results were expressed in Carratelli units (U.CARR); One U.CARR is equal to 0.08 mg/dl of H_2_O_2_. For BAP assay, the plasma was dissolved in a colored solution that was prepared previously by mixing FeCl_3_ with a thiocyanate derivative. After 5 min of incubation at 37 °C, the solution gradually lost its color, and the intensity was directly proportional to the ability of the plasma to reduce ferric ions to ferrous ions. Finally, the chromatic change was read at 505 nm.

### TUNEL staining and analysis

Paraffin-embedded brain sections were fixed at 24 h after ICH, then processed for terminal deoxynucleotidyl transferase-mediated dUTP-biotin nick end labeling assay (TUNEL; Takara, Tokyo, Japan) in accordance with the manufacturer’s protocol. Briefly, the sections were treated with proteinase K (20 μg/ml) for 20 min at room temperature, and then 50 μl of an FITC-labeling reaction mixture (consisting of TdT Enzyme 5 μl with FITC-Labeling Safe Buffer 45 μl) was applied to the slide and allowed to react for 90 min in a 37 °C humidified chamber. After that, the sections were counterstained with DAPI. TUNEL-positive cells were analyzed by fluorescent microscopy (n = 5). The number of TUNEL-positive cells under a high-power (x200) field was counted in four separate regions surrounding the hematoma (Fig. 2a), and the ratios of the positive cells to the total cell number were calculated and analyzed by computer. The counting was performed by an experimenter blinded to the sample information.

### Assessment of motor function (behavioral testing)

A grip strength test (GPM-100B; MELQUEST, Toyama, Japan) was performed before injury and at 6, 24 and 48 h after brain injury according to the manufacturer’s instructions. Briefly, the rats were held by their body, and allowed to grip the grid with the contralateral forelimb of the hemorrhaged side. Then, the rats were gently pulled downwards and the peak of the grip strength was recorded (in grams). Three trials were performed for each rat and the average was used as the animal’s forelimb grip force at that particular time point. For the forelimb use asymmetry test, forelimb use was analyzed by observing the rats in a transparent cylinder (20 cm in diameter and 40 cm in height) before and after ICH according to the method described previously^44^. All behavior tests were done by an experimenter blinded to the treatment.

### Statistical analysis

Statistical significance was evaluated using two-tailed Student’s *t*-test to compare two groups. For multiple comparisons, one-way ANOVA followed by the *post hoc* Bonferroni test or the *post hoc* Fisher’s LSD test was used. Excel Tohkei software (Social Survey Research Information, Tokyo, Japan) was used for the statistical analyses. A probability value of less than 0.05 was considered to indicate statistical significance. The results were expressed as the means ± SEM.

## Additional Information

**How to cite this article**: Wang, D. *et al*. Anti-high mobility group box-1 (HMGB1) antibody inhibits hemorrhage-induced brain injury and improved neurological deficits in rats. *Sci. Rep.* **7**, 46243; doi: 10.1038/srep46243 (2017).

**Publisher's note:** Springer Nature remains neutral with regard to jurisdictional claims in published maps and institutional affiliations.

## Supplementary Material

Supplementary Information

## References

[b1] Keep R. F., Hua Y. & Xi G. Intracerebral haemorrhage: mechanisms of injury and therapeutic targets. Lancet Neurol 11, 720–731 (2012). 22698888 10.1016/S1474-4422(12)70104-7 PMC3884550

[b2] Zhou Y., Wang Y., Wang J., Anne Stetler R. & Yang Q. W. Inflammation in intracerebral hemorrhage: from mechanisms to clinical translation. Prog Neurobiol 115, 25–44 (2014). 24291544 10.1016/j.pneurobio.2013.11.003

[b3] Katsuki H. Exploring neuroprotective drug therapies for intracerebral hemorrhage. Journal of pharmacological sciences 114, 366–378 (2010). 21081835 10.1254/jphs.10r05cr

[b4] Urday S. . Targeting secondary injury in intracerebral haemorrhage–perihaematomal oedema. Nature reviews. Neurology 11, 111–122 (2015). 25623787 10.1038/nrneurol.2014.264

[b5] Guan J. & Hawryluk G. W. Targeting Secondary Hematoma Expansion in Spontaneous Intracerebral Hemorrhage - State of the Art. Frontiers in neurology 7, 187 (2016). 27826284 10.3389/fneur.2016.00187 PMC5078502

[b6] Hanggi D. & Steiger H. J. Spontaneous intracerebral haemorrhage in adults: a literature overview. Acta Neurochir (Wien) 150, 371–379; discussion 379 (2008). 18176774 10.1007/s00701-007-1484-7

[b7] Liew H. K. . Systemic administration of urocortin after intracerebral hemorrhage reduces neurological deficits and neuroinflammation in rats. J Neuroinflammation 9, 13 (2012). 22257737 10.1186/1742-2094-9-13 PMC3271957

[b8] Keep R. F. . Vascular disruption and blood-brain barrier dysfunction in intracerebral hemorrhage. Fluids and barriers of the CNS 11, 18 (2014). 25120903 10.1186/2045-8118-11-18 PMC4130123

[b9] Urday S. . Measurement of perihematomal edema in intracerebral hemorrhage. Stroke 46, 1116–1119 (2015). 25721012 10.1161/STROKEAHA.114.007565 PMC5340416

[b10] Tsung A., Tohme S. & Billiar T. R. High-mobility group box-1 in sterile inflammation. Journal of internal medicine 276, 425–443 (2014). 24935761 10.1111/joim.12276

[b11] Liu K. . Anti-high mobility group box 1 monoclonal antibody ameliorates brain infarction induced by transient ischemia in rats. FASEB journal: official publication of the Federation of American Societies for Experimental Biology 21, 3904–3916 (2007). 17628015 10.1096/fj.07-8770com

[b12] Zhang J. . Anti-high mobility group box-1 monoclonal antibody protects the blood-brain barrier from ischemia-induced disruption in rats. Stroke 42, 1420–1428 (2011). 21474801 10.1161/STROKEAHA.110.598334

[b13] Okuma Y. . Anti-high mobility group box-1 antibody therapy for traumatic brain injury. Annals of neurology 72, 373–384 (2012). 22915134 10.1002/ana.23602

[b14] Sasaki T. . Anti-high mobility group box 1 antibody exerts neuroprotection in a rat model of Parkinson’s disease. Exp Neurol 275 Pt 1, 220–231 (2016). 26555088 10.1016/j.expneurol.2015.11.003

[b15] Nakamura Y. . Neuropathic pain in rats with a partial sciatic nerve ligation is alleviated by intravenous injection of monoclonal antibody to high mobility group box-1. PloS one 8, e73640 (2013). 23991202 10.1371/journal.pone.0073640 PMC3749159

[b16] van Zoelen M. A. . Role of toll-like receptors 2 and 4, and the receptor for advanced glycation end products in high-mobility group box 1-induced inflammation *in vivo*. Shock (Augusta, Ga.) 31, 280–284 (2009). 10.1097/SHK.0b013e318186262d PMC4535325 19218854

[b17] Ohnishi M. . HMGB1 inhibitor glycyrrhizin attenuates intracerebral hemorrhage-induced injury in rats. Neuropharmacology 61, 975–980 (2011). 21752338 10.1016/j.neuropharm.2011.06.026

[b18] Lei C. . HMGB1 may act via RAGE to promote angiogenesis in the later phase after intracerebral hemorrhage. Neuroscience 295, 39–47 (2015). 25813710 10.1016/j.neuroscience.2015.03.032

[b19] Lei C., Wu B., Cao T., Zhang S. & Liu M. Activation of the high-mobility group box 1 protein-receptor for advanced glycation end-products signaling pathway in rats during neurogenesis after intracerebral hemorrhage. Stroke 46, 500–506 (2015). 25538203 10.1161/STROKEAHA.114.006825

[b20] Wu H. . PGE 2 receptor agonist misoprostol protects brain against intracerebral hemorrhage in mice. Neurobiology of aging 36, 1439–1450 (2015). 25623334 10.1016/j.neurobiolaging.2014.12.029 PMC4417504

[b21] Xi G., Keep R. F. & Hoff J. T. Erythrocytes and delayed brain edema formation following intracerebral hemorrhage in rats. J Neurosurg 89, 991–996 (1998). 9833826 10.3171/jns.1998.89.6.0991

[b22] Zhou Y. . Elevation of high-mobility group protein box-1 in serum correlates with severity of acute intracerebral hemorrhage. Mediators of inflammation 2010 (2010). 10.1155/2010/142458 PMC2948906 20936104

[b23] Lei C. . High-mobility group box1 protein promotes neuroinflammation after intracerebral hemorrhage in rats. Neuroscience 228, 190–199 (2013). 23085216 10.1016/j.neuroscience.2012.10.023

[b24] Su X., Wang H., Zhao J., Pan H. & Mao L. Beneficial effects of ethyl pyruvate through inhibiting high-mobility group box 1 expression and TLR4/NF-kappaB pathway after traumatic brain injury in the rat. Mediators of inflammation 2011, 807142 (2011). 21772666 10.1155/2011/807142 PMC3136093

[b25] Li D. . Blockade of high mobility group box-1 signaling via the receptor for advanced glycation end-products ameliorates inflammatory damage after acute intracerebral hemorrhage. Neurosci Lett 609, 109–119 (2015). 26483322 10.1016/j.neulet.2015.10.035

[b26] Zhang F. F. . Perineural expression of high-mobility group box-1 contributes to long-lasting mechanical hypersensitivity via matrix metalloproteinase-9 upregulation in mice with painful peripheral neuropathy. Journal of neurochemistry(2015). 10.1111/jnc.13434 26578177

[b27] Yu M. . HMGB1 signals through toll-like receptor (TLR) 4 and TLR2. Shock (Augusta, Ga.) 26, 174–179 (2006). 10.1097/01.shk.0000225404.51320.82 16878026

[b28] Hori O. . The receptor for advanced glycation end products (RAGE) is a cellular binding site for amphoterin. Mediation of neurite outgrowth and co-expression of rage and amphoterin in the developing nervous system. The Journal of biological chemistry 270, 25752–25761 (1995). 7592757 10.1074/jbc.270.43.25752

[b29] Yang F. . Receptor for advanced glycation end-product antagonist reduces blood-brain barrier damage after intracerebral hemorrhage. Stroke 46, 1328–1336 (2015). 25782468 10.1161/STROKEAHA.114.008336

[b30] Taylor R. A. & Sansing L. H. Microglial responses after ischemic stroke and intracerebral hemorrhage. Clinical & developmental immunology 2013, 746068 (2013). 24223607 10.1155/2013/746068 PMC3810327

[b31] Sha Y., Zmijewski J., Xu Z. & Abraham E. HMGB1 develops enhanced proinflammatory activity by binding to cytokines. Journal of immunology (Baltimore, Md.: 1950) 180, 2531–2537 (2008). 10.4049/jimmunol.180.4.2531 18250463

[b32] Minghetti L. Cyclooxygenase-2 (COX-2) in inflammatory and degenerative brain diseases. Journal of neuropathology and experimental neurology 63, 901–910 (2004). 15453089 10.1093/jnen/63.9.901

[b33] Giulian D. & Lachman L. B. Interleukin-1 stimulation of astroglial proliferation after brain injury. Science (New York, N.Y.) 228, 497–499 (1985). 10.1126/science.3872478 3872478

[b34] Allan S. M., Tyrrell P. J. & Rothwell N. J. Interleukin-1 and neuronal injury. Nat Rev Immunol 5, 629–640 (2005). 16034365 10.1038/nri1664

[b35] Chu K. . Celecoxib induces functional recovery after intracerebral hemorrhage with reduction of brain edema and perihematomal cell death. J Cereb Blood Flow Metab 24, 926–933 (2004). 15362723 10.1097/01.WCB.0000130866.25040.7D

[b36] Rosenberg G. A. & Navratil M. Metalloproteinase inhibition blocks edema in intracerebral hemorrhage in the rat. Neurology 48, 921–926 (1997). 9109878 10.1212/wnl.48.4.921

[b37] Power C. . Intracerebral hemorrhage induces macrophage activation and matrix metalloproteinases. Annals of neurology 53, 731–742 (2003). 12783419 10.1002/ana.10553

[b38] Moynagh P. N. The interleukin-1 signalling pathway in astrocytes: a key contributor to inflammation in the brain. Journal of anatomy 207, 265–269 (2005). 16185251 10.1111/j.1469-7580.2005.00445.x PMC1571539

[b39] Ferrari C. C. . Reversible demyelination, blood-brain barrier breakdown, and pronounced neutrophil recruitment induced by chronic IL-1 expression in the brain. The American journal of pathology 165, 1827–1837 (2004). 15509551 10.1016/S0002-9440(10)63438-4 PMC1618664

[b40] Haruma J. . Anti-high mobility group box-1 (HMGB1) antibody attenuates delayed cerebral vasospasm and brain injury after subarachnoid hemorrhage in rats. Scientific reports 6 (2016). 10.1038/srep37755 PMC5121891 27883038

[b41] Wasserman J. K. & Schlichter L. C. Minocycline protects the blood-brain barrier and reduces edema following intracerebral hemorrhage in the rat. Exp Neurol 207, 227–237 (2007). 17698063 10.1016/j.expneurol.2007.06.025

[b42] Park H. K. . Granulocyte colony-stimulating factor induces sensorimotor recovery in intracerebral hemorrhage. Brain research 1041, 125–131 (2005). 15829221 10.1016/j.brainres.2004.11.067

[b43] Wang J. & Dore S. Heme oxygenase-1 exacerbates early brain injury after intracerebral haemorrhage. Brain 130, 1643–1652 (2007). 17525142 10.1093/brain/awm095 PMC2291147

[b44] Okuma Y. . Glycyrrhizin inhibits traumatic brain injury by reducing HMGB1-RAGE interaction. Neuropharmacology 85, 18–26 (2014). 24859607 10.1016/j.neuropharm.2014.05.007

