# Supplementary material for: Anti-high mobility group box-1 (HMGB1) antibody inhibits hemorrhage-induced brain injury and improved neurological deficits in rats
Source: Sci Rep. 2017 Apr 10;7:46243. doi: 10.1038/srep46243 (PMC5385548; doi:10.1038/srep46243)
Supplement: Supplementary Information [file srep46243-s1.pdf]

ONLINE SUPPLEMENT

Supplementary Tables and Figures

Anti-high mobility group box-1 (HMGB1) antibody inhibits hemorrhage-induced brain injury and improved neurological deficits in rats

Dengli Wang[1], Keyue Liu[1], Hidenori Wake[1], Kiyoshi Teshigawara[1], Shuji Mori[2] and Masahiro Nishibori[1]*

[1]Department of Pharmacology, Okayama University Graduate School of Medicine, Dentistry and Pharmaceutical Sciences, Okayama, Japan

[2]Shujitsu University, School of Pharmacy, Okayama, Japan

Correspondence to

Masahiro Nishibori, MD, PhD.

Department of Pharmacology, Okayama University Graduate School of Medicine, Dentistry and Pharmaceutical Sciences, 2-5-1 Shikata-cho, Kita-ku, Okayama 700-8558, Japan. Tel/FAX: +81-86- 235-7140.

E-mail: mbori@md.okayama-u.ac.jp

Supplementary table S1: Primer Sequences Used in quantitative RT-PCR

Gene	
iNOS	5'-GCATCCCAAGTACGAGTGGT-3'
	5'-GAAGTCTCGGACTCCAATCTC-3'
TNF-a	5'-GCCCAGACCCTCACACTC-3'
	5'-CCACTCCAGCTGCTCCTCT-3'
MMP-2	5'-GCACCGTCGCCCATCA-3'
	5'-GTCTCGATGGTGTTCTGGTCAA-3'
MMP-9	5'-GAGGATCCGCAGTCCAAGAA-3'
	5'-GCACCGTCTGGCCTGTGTA-3'
COX-2	5'-TGTATGCTACCATCTGGCTTCGG-3'
	5'-GTTTGGAACAGTCGCTCGTCATC-3'
IL-6	5'-CAAAGCCAGAGTCATTCAGAGC-3'
	5'-GGAGAGCATTGGAAGTTGGG-3'
VEGF-121	5'-CTCACCAAAGCCAGCACATA-3'
	5'-GCCTTGGCTTGTCACATT-3'
IL-8R	5'-CATCCTGCCTCAGACCTATGG-3'
	5'-AAGACGAGGACCACAGCAAAG-3'
GAPDH	5'-AGCCCAGAACATCATCCCTG-3'
	5'-CACCACCTTCTTGATGTCATC-3'
IL-1β	5'-CACCTTCTTTTCCTTCATCTTTG-3'
	5'-GTCGTTGCTTGTCTCTCCTTGTA-3'
AT-1R	5′-ACCAGGTCAAGTGGATTTCG-3′
	5′-ATCACCACCAAGCTGTTTCC-3′
PAR-1	5′-GTTGGATAGTGGGCCGTAGA-3′
	5′-TTAGCTGATAGGCCGTGCTT-3′
α-1R	5′-GAATGTCCTGCGAATCCAGT-3
	5′-GATTGGTCCTTTGGCACTGT-3′
V1R	5′-GGTCGTCTTGGGTACATGCT-3′
	5′-TCTTCACAGTGCGGATCTTG-3′
TxA2	5′-AGGAGCCTGAATGTTTGGTG-3′
	5′-TGAGACAGACGCGGACTATG-3′
eNOS	5′-TGACCCTCACCGATACAACA-3′
	5′-CTGGCCTTCTGCTCATTTTC-3′
IL-10	5′-CCTCTGGATACAGCTGCGAC-3′
	5′-CATTCATGGCCTTGTAGACACC-3′
HMGB1	5′-TTGTCCACACACCCTGCATA-3′
	5′-AATTGATCACTCCTTGCTTTGCT-3′
TLR2	5′-GACTCAAGAGCATCGGCTGG-3′
	5′-CAGAATGGCCTTCCCTTGAGA-3′
TLR4	5′-CTCACAACTTCAGTGGCTGG-3′
	5′-GGGTTTCCTGTCAGTACCAAGG-3′
RAGE	5′-CTGAGGTAGGGCATGAGGATG-3′
	5′-GCCTGCAGCTTGTCCTTCAT-3′

Suppl. S1a

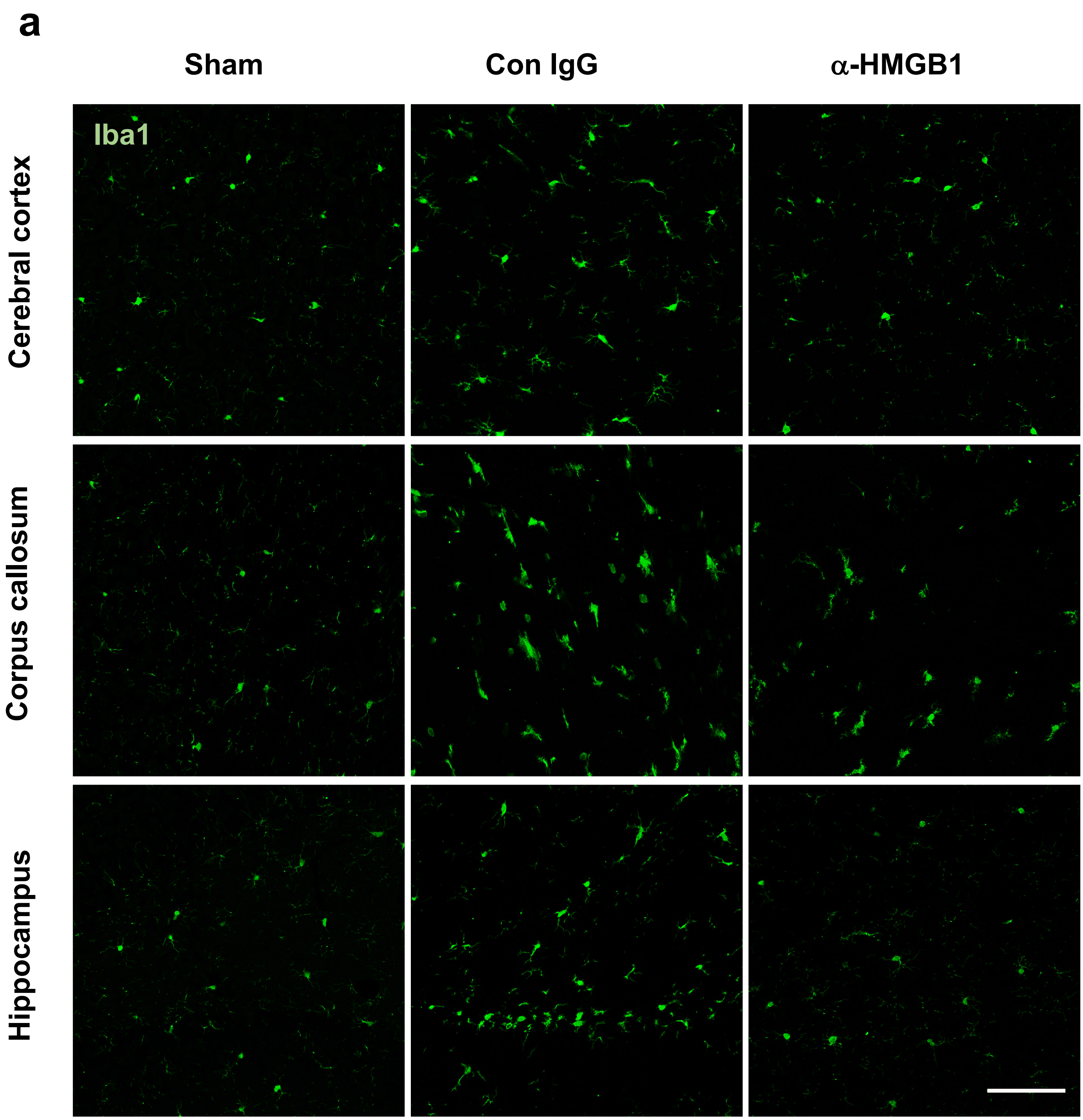

Supplementary Fig. S1a: The expression of Iba1 in the cerebral cortex, corpus callosum and hippocampus 24 h after ICH. The Iba1-immunoreactive cells were observed in the cerebral cortex, corpus callosum, striatum (perihematomal region) and hippocampus 24 h after ICH. A representative image from 3-6 rats is shown in each group. The scale bar represents 100 μm.

Suppl. S1b

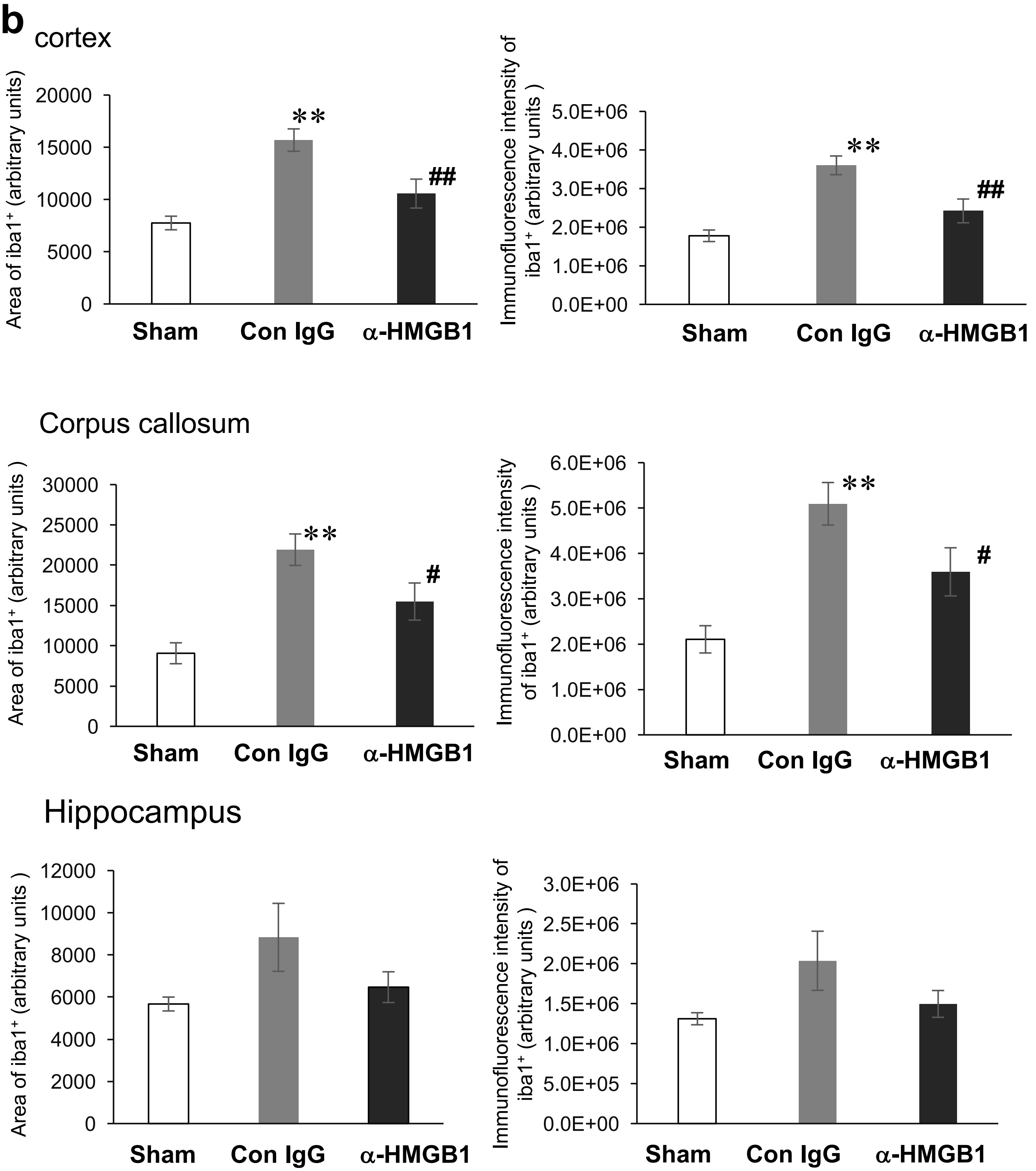

b
cortex
Area of iba1⁺ (arbitrary units)
20000
15000
10000
5000
0
**
##
Sham
Con IgG
α-HMGB1
Immunofluorescence intensity of iba1⁺ (arbitrary units)
5.0E+06
4.0E+06
3.0E+06
2.0E+06
1.0E+06
0.0E+00
**
##
Sham
Con IgG
α-HMGB1
Corpus callosum
Area of iba1⁺ (arbitrary units)
30000
25000
20000
15000
10000
5000
0
**
#
Sham
Con IgG
α-HMGB1
Immunofluorescence intensity of iba1⁺ (arbitrary units)
6.0E+06
5.0E+06
4.0E+06
3.0E+06
2.0E+06
1.0E+06
0.0E+00
**
#
Sham
Con IgG
α-HMGB1
Hippocampus
Area of iba1⁺ (arbitrary units)
12000
10000
8000
6000
4000
2000
0
Sham
Con IgG
α-HMGB1
Immunofluorescence intensity of iba1⁺ (arbitrary units)
3.0E+06
2.5E+06
2.0E+06
1.5E+06
1.0E+06
5.0E+05
0.0E+00
Sham
Con IgG
α-HMGB1

Supplementary Fig. S1b: Quantification of immunoreactive area and immunofluorescence intensity for Iba1. The immunoreactive area for Iba1 and immunofluorescence intensity at a constant size were measured in each rat and the results are expressed as the means ± SEM of 4-6 rats. $F_{(2,11)}$=4.996, p=0.029; $F_{(2,11)}$ =5.426, p=0.023 for cortex. F $_{(2,13)}$ =12.607, p<0.001; F $_{(2,13)}$ =12.448, p<0.001for corpus callosum. F $_{(2,10)}$ =1.819, p=0.212; F $_{(2,10)}$ =1.811, p=0.213 for hippocampus. **p < 0.01 compared with the sham groups. $^{\#}$p < 0.01, $^{\#}$p < 0.05 compared with the control IgG-treated group.

Suppl. S2

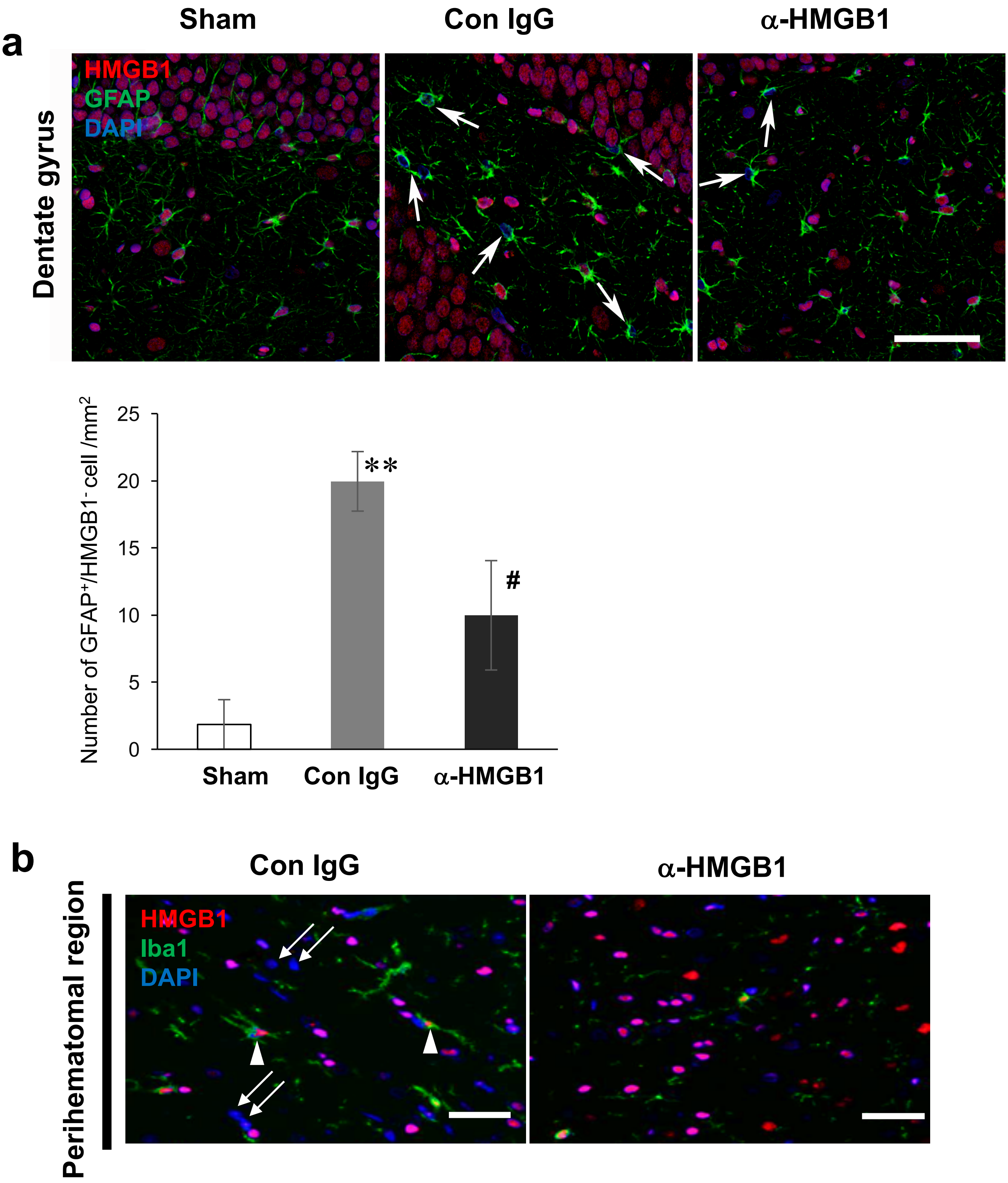
a
Sham
Con IgG
α-HMGB1
Dentate gyrus
HMGB1
GFAP
DAPI
Number of GFAP+/HMGB1- cell /mm2
25
20
15
10
5
0
**
#
Sham
Con IgG
α-HMGB1
b
Con IgG
α-HMGB1
Perihematomal region
HMGB1
Iba1
DAPI

Supplementary Fig. S2: Double immunohistochemical staining for HMGB1 and GFAP in the dentate gyrus and immunofluorescence experiments of microglia activation and HMGB1 expression in the ICH brain. a Double immunohistochemical staining for HMGB1 and GFAP in the dentate gyrus was performed 24 h after ICH. The white arrows indicate HMGB1-negative astrocytes. The scale bars represent 50 μm. The number of these cells was counted in each group and the results are expressed as the means ± SEM of 3-5 rats. $F_{(2,10)}=7.168$, $p=0.012$. **p < 0.01 compared with the sham groups. #p < 0.05 compared with the control IgG-treated group. **b** Activated microglia in the peri-hematomal region was detected by Iba1 staining. White arrowheads show Iba1-strong positive activated microglia. Arrows indicate the DAPI-positive but HMGB1-negative cells. The scale bar represents 50 μm.

Suppl. S3

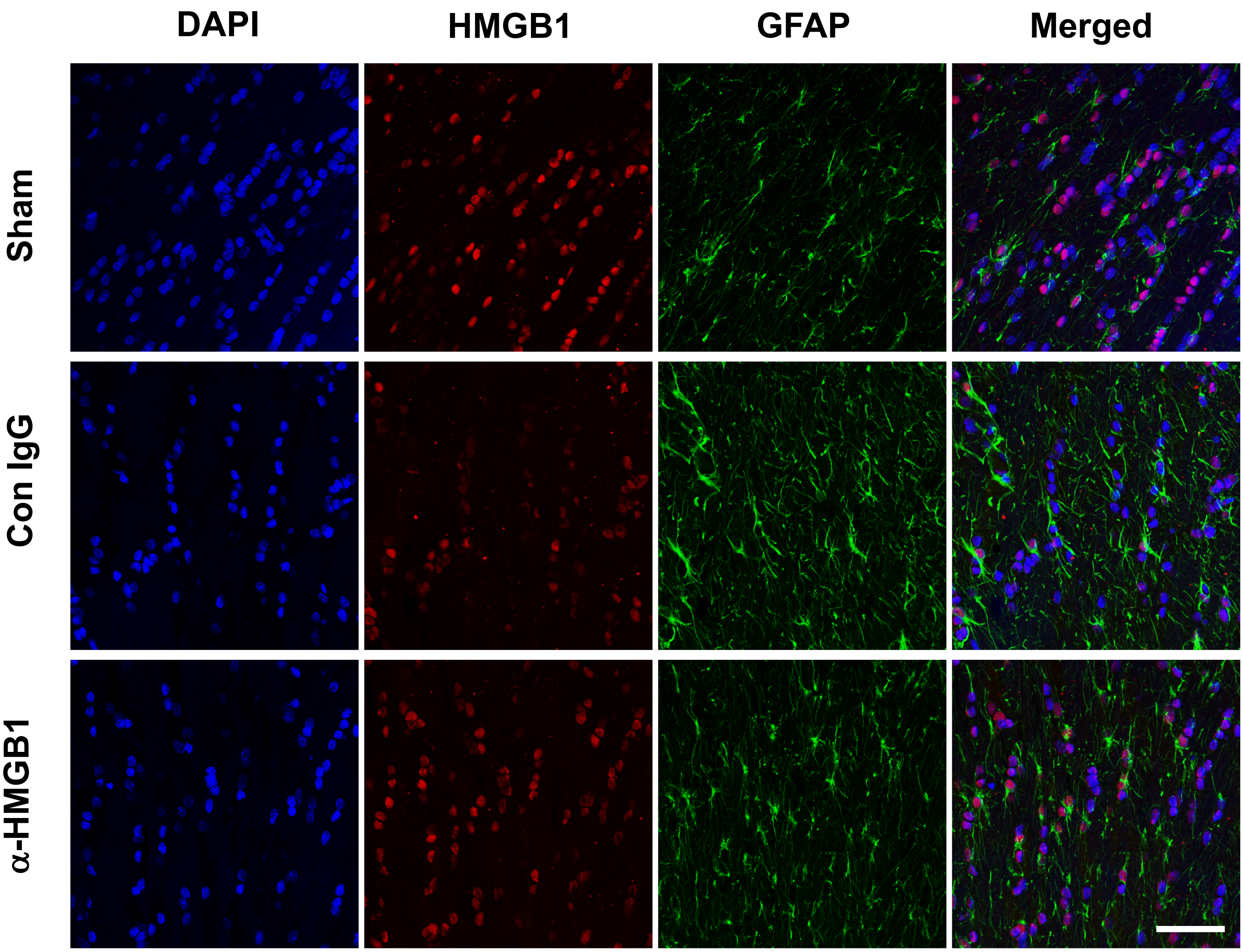

DAPI
HMGB1
GFAP
Merged
Sham
Con IgG
α-HMGB1

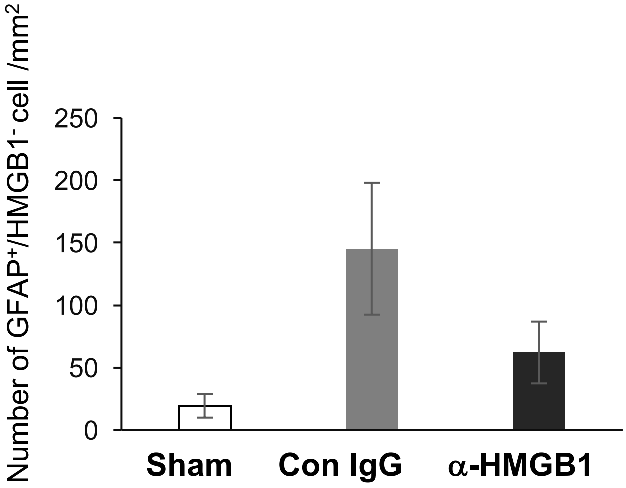

Number of GFAP+/HMGB1- cell /mm2
250
200
150
100
50
0
Sham
Con IgG
α-HMGB1

Supplementary Fig. 3 The translocation of HMGB1 from astrocytes in the corpus callosum 24 h after ICH. Double immunohistochemical staining for HMGB1 and GFAP in the corpus callosum was performed 24 h after ICH. Most of the GFAP-positive astrocytes in the control IgG group were negative for HMGB1, whereas those in the anti-HMGB1 group retained HMGB1 in their nuclei. The scale bar represents 50 μm. The ratio of HMGB1-negative astrocytes to total astrocytes in corpus callosum are expressed as the means ± SEM of 4-5 rats. $F_{(2,11)}$ =2.988, p=0.092.

Suppl. S4

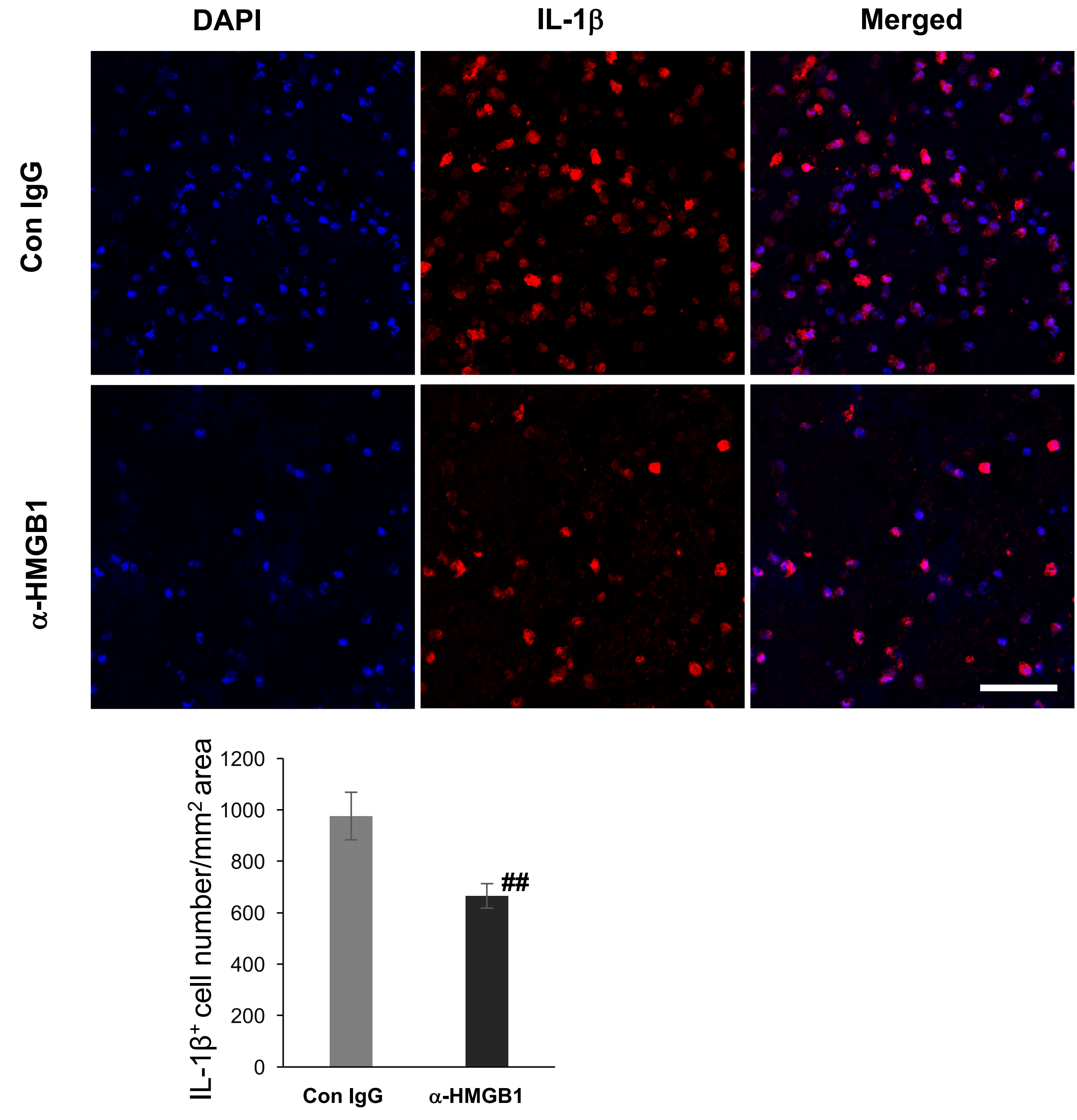

Supplementary Fig. S4 Effect of anti-HMGB1 on IL-1β immunoreactivities within the hemorrhagic region at 24 h after ICH. IL-1β immunoreactivities were detected within the hemorrhagic region at 24 h after ICH. The scale bars represent 50 μm. The total cell number was counted in two groups and the results are expressed as the means ± SEM of 5 rats. $^{\#\#}p < 0.01$ compared to the control IgG group.

Suppl. S5

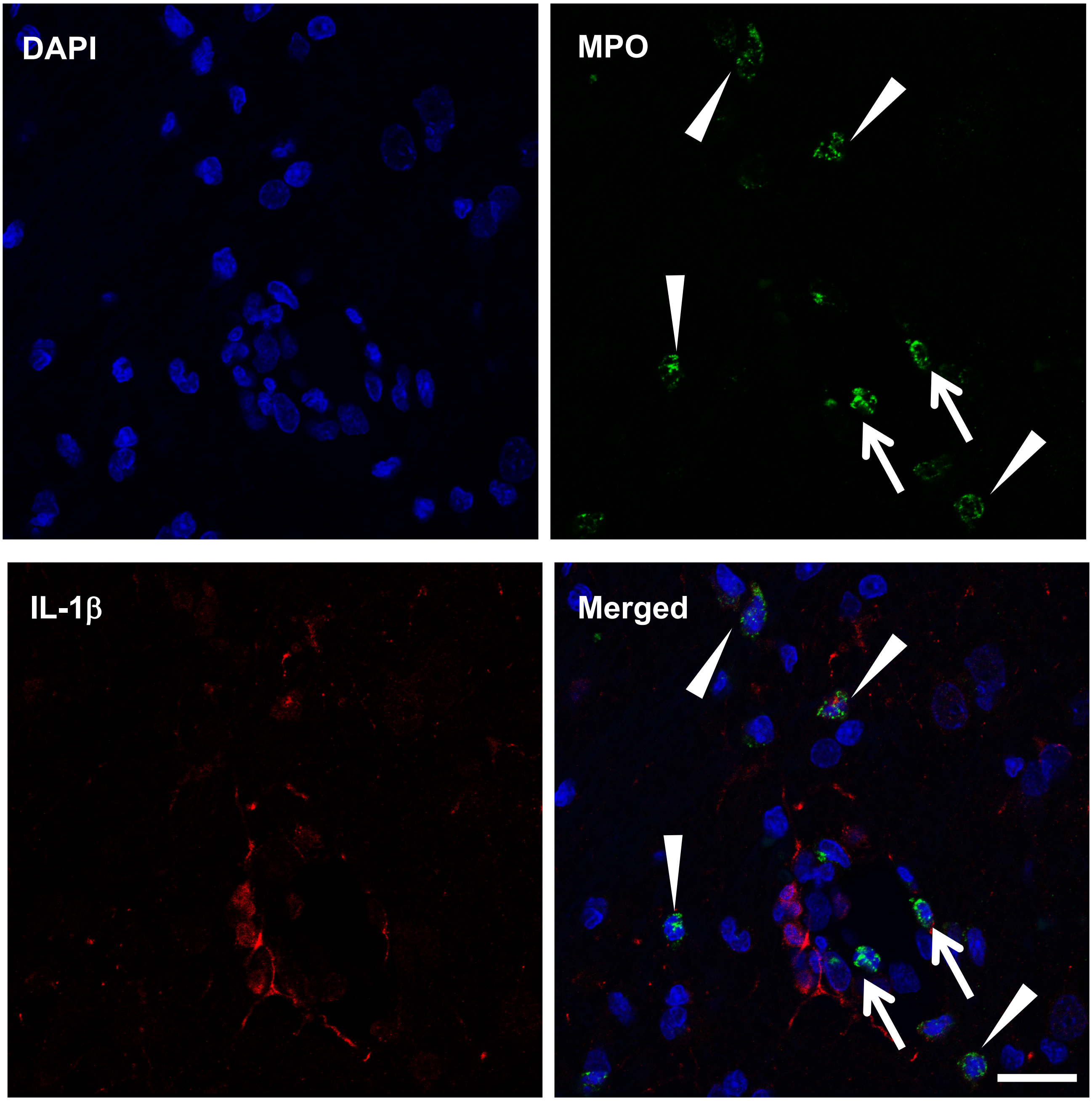

Supplementary Fig. S5: A representative image of a vessel in the perihematomal region. MPO^+ cells existed inside and outside the blood vessel. IL-1β^+ astrocytic processes surrounding and embracing the blood vessel. Arrows indicate the MPO^+ cells inside the vessel. Arrowheads indicate MPO^+ cells outside the vessel. The scale bar represents 20 μm.

Suppl. S6

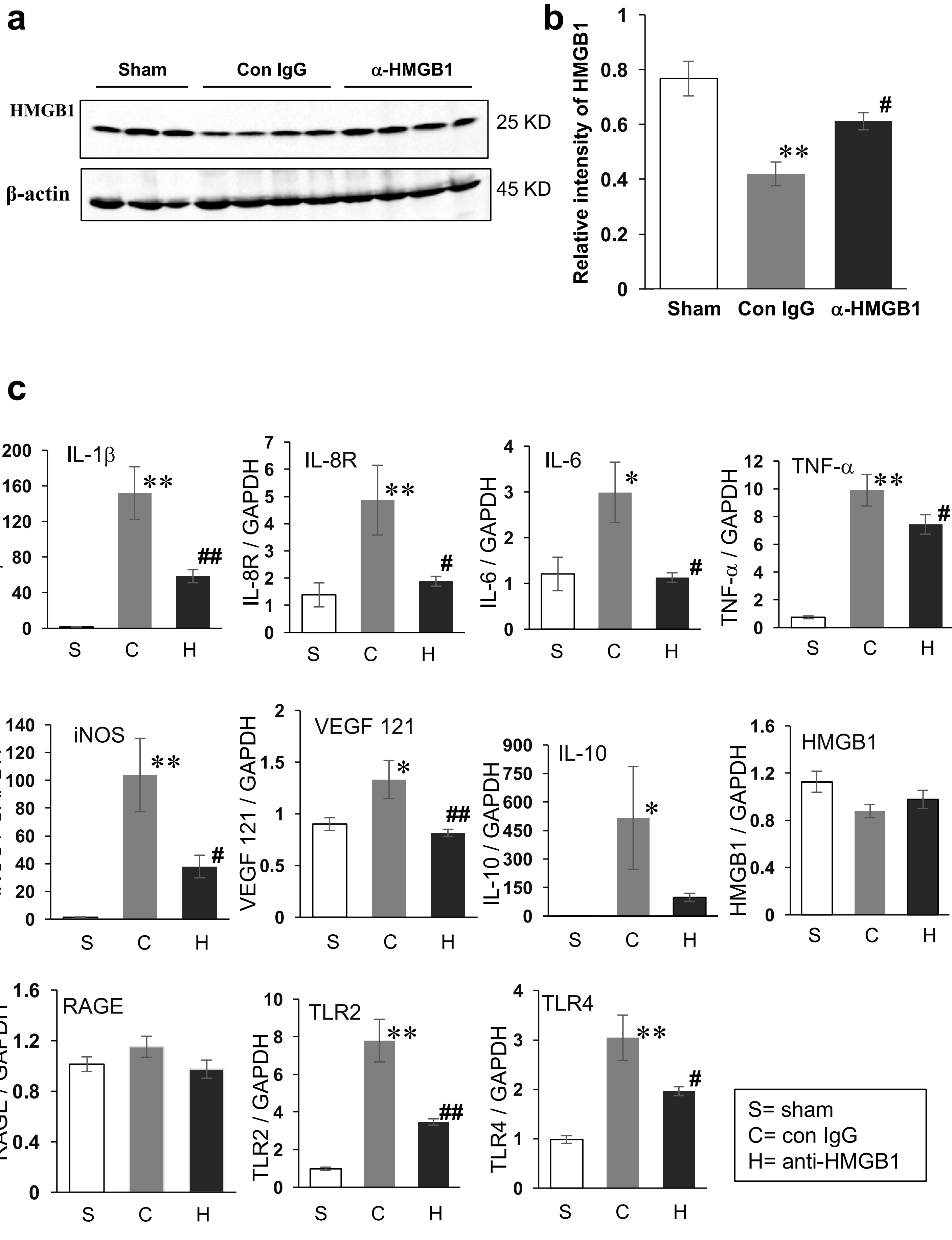

a
Sham
Con IgG
α-HMGB1
HMGB1
25 KD
β-actin
45 KD
b
Relative intensity of HMGB1
Sham
Con IgG
α-HMGB1
c
IL-1β
IL-1β / GAPDH
IL-8R
IL-8R / GAPDH
IL-6
IL-6 / GAPDH
TNF-α
TNF-α / GAPDH
iNOS
iNOS / GAPDH
VEGF 121
VEGF 121 / GAPDH
IL-10
IL-10 / GAPDH
HMGB1
HMGB1 / GAPDH
RAGE
RAGE / GAPDH
TLR2
TLR2 / GAPDH
TLR4
TLR4 / GAPDH
S= sham
C= con IgG
H= anti-HMGB1

Supplementary Fig. S6 Evaluation of the effects of anti-HMGB1 mAb treatment with regard to the therapeutic time window. The treatment of anti-HMGB1 (1mg/kg) or control mAb was given at both 3 h and 6 h after ICH induction. **a** Cerebral bleeding areas with a volume of 3 x 3 x 3 mm^3 (as indicated by the white square in Fig1a) were sampled at 24 h after ICH for western blotting to determine brain HMGB1 levels. The representative results of western blotting are shown. **b** Quantitative analyses of the western blotting results were performed using NIH Image J software. The results are expressed as the means ± SEM of 3-4 rats. F(2,8)=14.589, p=0.002 **p < 0.01 compared with the sham group. #p < 0.05 compared with the control IgG-treated group. **c** mRNA expression was measured by quantitative real-time PCR in the ipsilateral (injured) striatum at 24 h after ICH. F value for each result was shown below as IL-1β ($F_{(2,12)}$=18.557, p<0.001), IL-8R ($F_{(2,12)}$=5.682, p=0.018), TNF-α ($F_{(2,12)}$=37.675, p<0.001), iNOS ($F_{(2,12)}$=10.630, p=0.002), IL-6 ($F_{(2,12)}$=5.713, p=0.018), VEGF121 ($F_{(2,12)}$=5.875, p=0.017) RAGE ($F_{(2,12)}$=1.6762, p=0.228), TLR2 ($F_{(2,12)}$=26.971, p<0.001), TLR4 ($F_{(2,12)}$=14.136, p<0.001), IL-10: ($F_{(2,12)}$=3.204, p=0.077) HMGB1 ($F_{(2,12)}$=2.795, p=0.101). The results are expressed as the means ± SEM of 5 rats. *p < 0.05, **p < 0.01 compared with the sham groups. #p < 0.05, ##p < 0.01 compared with the control IgG-treated group.
